# Active transcutaneous bone conduction hearing implants: Systematic review and meta-analysis

**DOI:** 10.1371/journal.pone.0221484

**Published:** 2019-09-16

**Authors:** Astrid Magele, Philipp Schoerg, Barbara Stanek, Bernhard Gradl, Georg Mathias Sprinzl

**Affiliations:** 1 University Clinic St. Poelten, Department of Otorhinolaryngology, Head & Neck Surgery, St. Poelten, Austria; 2 Karl Landsteiner Institute of Implantable Hearing Devices, St. Poelten, Austria; University of Miami School of Medicine, UNITED STATES

## Abstract

**Background:**

In July 2018 the active transcutaneous bone conduction hearing implant received FDA approval in the US (for patients 12 years and older with conductive and/or mixed hearing loss or single-sided deafness), reflecting the current trend of moving away from percutaneous hearing solutions towards intact skin systems.

**Objectives:**

To critically assess the current literature on safety, efficacy and subjective benefit after implantation with an active transcutaneous bone conduction hearing device.

**Data sources:**

Literature investigation was performed by electronic database search including PubMed and Cochrane Central Register of Controlled Trials, and manual search of relevant journals and reference lists of included studies.

**Study eligibility criteria:**

Randomized controlled trials, clinical controlled trials and cohort studies, case series and case reports investigating subjective and objective outcomes.

**Study appraisal and synthesis methods:**

Retrieved literature was screened and extracted by two reviewers independently. Subgroup analysis of indications (conductive and/or mixed hearing loss, single-sided deafness) and participant ages (pediatric vs. adults) was conducted on patients with active transcutaneous bone conduction devices. Sensitivity analysis was performed to test the stability of the results in meta-analysis.

**Results:**

39 citations reporting on pre- and postoperative audiological results, speech performance in quiet and in noise, localization testing as well as subjective outcomes were included in this systematic review. Functional gain as well as word recognition score outcomes could be further investigated via meta-analysis. All outcomes reported and summarized here reflect beneficial audiological performance and high patient satisfaction, accompanied with a low complications rate (minor event incidence rate: 9.9 person-years; major incidence rate: 148.9 person-years) for the indications of conductive and mixed hearing loss as well as in individuals suffering from single-sided deafness for all age groups of subjects who underwent active transcutaneous bone conduction hearing device implantation.

**Limitations:**

A limiting factor of this systematic review was the Level of Evidence of the reviewed literature, comprising 2a/3a studies (cohort studies and case-control studies). Furthermore, the reporting standards, especially in outcomes such as word recognition scores in quiet and in noise, vary across study cites from various countries, which impedes comparisons. Last but not least, no other comparable other device was retrieved as the active transcutaneous bone conduction hearing device is the only available at the moment.

**Conclusion:**

The device’s transcutaneous technology results in a minor event incidence rate of one in 9.9 person-years and a major incidence rate of one in 148.9 person-years. Based on the audiological outcomes, high patient satisfaction as well as the low complication rate, the authors recommend the active transcutaneous bone conduction hearing device as a safe and effective treatment for patients suffering from hearing loss within the device’s indication criteria (conductive and/or mixed hearing loss or single-sided deafness).

## Introduction

In 2018, the WHO reported that around 466 million people worldwide (34 million children) suffer from disabling hearing loss, defined as hearing loss greater than 40dB in the better hearing ear in adults and 30dB in the better hearing ear in children. Advances in medicine and technology have led to many new treatment options for all different types as well as severities of hearing loss and include hearing aids, medical intervention via prostheses and surgically implanted medical devices such as cochlear, middle ear, or, as currently reviewed, bone conduction implants. While the majority of patients with moderate to severe hearing loss can be supplied with conventional hearing aids, some patients either do not benefit enough from hearing aids or cannot wear them due to anatomical or skin-related issues. In these cases, implantable hearing devices fill a clinical need and active transcutaneous bone conduction implants (atBCI) may serve as a valuable solution for adults and children with moderate to severe conductive (CHL) and mixed hearing loss (MHL), as well as those affected by single-sided deafness (SSD). Surgical and technical details as well as information about the device’s indications have been previously published by the authors [1]. The first atBCI, namely the Bonebridge (MED-EL, Austria) was implanted in June 2011 as part of a clinical trial. Following completion of the clinical trial, market approval and a controlled market entry, the atBCI was launched EU-wide in September 2012 and in further countries shortly thereafter. After receiving the CE marking (certification mark regarding conformity within the European Economic Area) in 2012 for adults (>18 years), in 2014 the indication was extended to children over the age of five years. In July 2018, the implant received FDA approval in the US (for patients 12 years and older with conductive and/or mixed hearing loss (C/MHL) or SSD), reflecting the current trend of moving away from percutaneous hearing solutions towards intact skin systems. Six years after its initial launch, the atBCI is being implanted in more than 200 centres all over the world, and a vast amount of literature has been published reporting on its efficacy, safety and effectiveness in clinical routine in more or less controlled case series and case reports. These types of studies often lead to potentially biased conclusions about the device’s performance, as the evidence is not comprised of high-quality study designs such as randomized clinical trials. Although the application of such types of studies can be difficult, if not impossible to pursue in clinical application, the introduced bias needs to be carefully addressed when drawing conclusions on the overall performance of a device or treatment. Therefore, meta-analysis models were selected to properly reflect the combined study outcomes on audiological (WRS at 65dB and functional gain) as well as safety outcomes with the active transcutaneous bone conduction implant. A meta-analysis integrates the quantitative findings from separate but similar studies and provides a numerical estimate of the overall effect of interest. Searches were conducted based on specifically identified PICOS: **Population**—Subjects of any age, gender or ethnicity. **Intervention**—Implantation of the active transcutaneous bone conduction implant by either surgical approach. **Comparators**—n/a. **Outcomes**—Data regarding safety, efficacy, quality of life and subjective outcomes with the device. Efficacy outcomes were divided into audiological/performance outcomes, including preoperative and postoperative hearing thresholds, functional gain, speech perception in quiet and noise, speech recognition thresholds, sound localisation; and subjective outcomes determined by questionnaires, patient-oriented scales of improvement and satisfaction scales. **Study design**—All study designs were included. Letters, editorials and systematic reviews with no original data, animal, in-vitro and laboratory studies were excluded.

For the first time, a meta-analysis was conducted using the systematically-reviewed literature on the only active transcutaneous bone conduction implant in order to combine and compare data from multiple sources of similar methodological and scientific quality. This helps to provide a clearer picture of the effect of the intervention, as well as assisting clinicians in forming their opinions and giving recommendations about the treatment.

## Methods

Studies were searched based on previously identified PICOS (Table 1).

**Table 1:** Search terms and outcomes.

Search Steps	Search Terms	Hits
1	active transcutaneous bone conduction device OR atBCI OR atBCI *	69
2	(bone conduction device OR bone conduction device*) OR (bone anchored hearing aid OR bone anchored hearing aid*) OR (bone conduction hearing aid OR bone conduction hearing aid*) OR (bone conduction implant OR bone conduction implant*) OR (bone anchored hearing implant OR bone anchored hearing implant*) OR (conductive hearing aid OR conductive hearing aid*) OR (bone conduction hearing system OR bone conduction hearing system*)	2791
3	(BCI OR BCD OR BCHA OR BAHA OR BAHS OR BAHI) AND hearing aid*	507
4	#1 OR #2 OR #3	2829
5	Limit #4 to *Humans*	2216
	Filters: Publication date from 2012/01/01 to 2018/10/31	655

*Note*. The different search terms are connected using Boolean logic. Activated filters are displayed in *italics*.

* Wildcard symbol to broaden the search by creating a root word search.

Using the guidelines available from the Cochrane Collaboration, a search strategy and review protocol was developed (Table 1) using PubMed (MEDLINE) and Cochrane databases to identify all publications on the active transcutaneous bone conduction implant from 2012 to October 31^st^, 2018. These dates were set in accordance with the publication dates of the first known articles on the device.

Study selection and data extraction was performed after removing duplicates, with titles and abstracts screened against the set interface to conic and integer programming solvers (Table 2).

**Table 2:** Inclusion and Exclusion criteria for retrieved literature.

**Inclusion Criteria**	
Population	Subjects of any age, gender or ethnicity, unilateral or bilateral mixed or conductive hearing loss or single-sided deafness
Intervention/ treatment	active transcutaneous bone conduction device; atBCI
Comparator	Other treatment options for CHL, MHL or SSD, or no treatment directly compared within the study (ie.: BAHA (Cochlear), bone anchored hearing aids, the CROS, and Bone Conduction Hearing aids (Soft- and Headband)). *Cochlear implants were excluded*
Outcomes	Performance (efficacy), safety, quality of life, subjective outcomes
Study design	Randomized or nonrandomized comparative studies, case series, case-control studies, controlled/not controlled before and after studies and interrupted time series analyses. *Letters*, *editorials and systematic reviews with no original data*, *animal*, *in-vitro and laboratory studies were excluded*.
**Exclusion Criteria**	
	Different device or treatment Not a clinical study in humans Other type of hearing loss (not CHL, MHL or SSD) Neither safety nor performance or quality of life data reported Topic not related to hearing loss or treatment thereof Publication lacking sufficient information for evaluation OVERLAP OF DATA

Unrelated titles were removed, and the full texts of the remaining articles were obtained for further screening. Studies were excluded if they still did not fulfil the eligibility criteria or if appointed a negative quality rating. Two reviewers screened the full texts, who resolved any discrepancies with discussion. When required, data were estimated from figures, or mean and standard deviations were calculated from tables. In case of inconclusive or missing outcomes, the authors were contacted via mail and asked for clarification. Data extraction was performed using an adaption/extension of the Cochrane Review Data Extraction Template: outcomes taken from full texts were pre-operative freefield and soundfield hearing thresholds (air conduction (AC) as well as bone conduction (BC)), postoperative functional gain (unaided/aided outcomes), outcomes in word recognition scores (WRS) and if necessary benefit was calculated (especially for WRS at 65dB), as well as sound perception in noise and sound localization abilities. The incidence rates of adverse events were recorded and grouped into major and minor adverse events rates. Studies were excluded if overlapping samples were seen, or they were found to be of low quality (i.e. non-peer-reviewed publications such as proceedings and abstracts). In studies with overlapping samples, the study with the higher number of participants was used for analyses (for example, Bravo-Torres et al. 2017 (n = 15) was excluded due to subject overlap in Der et al. 2018 (n = 24)). To evaluate methodological quality and scientific validity, the full texts of the included literature were appraised according to the standard rating system (MEDDEV 2.7/1 rev. 4, Table 3).

**Table 3:** Literature appraisal criteria.

Data Suitability	Description	Grading System
Appropriate Device Application	Was the device used for the same intended use (e.g. methods of deployment, application, etc.)?	Same use
		Minor deviation
		Major deviation
Acceptable Report/Data Collation	Did the reports or collations of data contain sufficient information to be able to undertake a rational and objective assessment?	High quality
		Minor deficiencies
		Insufficient information
Data Contribution	Description	Grading System
Data Source Type	Was the design of the study appropriate?	Yes
		No
Outcome Measures	Did the outcome measures reported reflect the intended performance of the device?	Yes
		No
Follow-Up	Was the duration of the follow-up long enough to assess treatment effects and identify complications?	Yes
		No
Statistical Significance	Was a statistical analysis of the data provided and appropriate?	Yes
		No
Clinical Significance	Was the magnitude of the treatment effect observed clinically significant?	Yes
		No

Additionally the hierarchy of evidence was graded using the Oxford level of evidence chart (http://www.cebm.net/index.aspx?o=5653). Furthermore, possible conflicts of interest which could lead to bias were evaluated and appraisal outcomes were summarized. The extracted outcomes were assessed, if possible, via meta-analysis within the R Statistical Computing Environment using the metafor package [2–4]. More specifically, separate random effect models were fitted to the following outcome variables: 1) mean functional gain (FG), 2) mean benefit in word recognition score at 65dB (WRS), and follow-up-dependent incidence rates in person years were calculated for 3) minor adverse events and 4) major adverse events. For audiological outcomes (FG and WRS), separate models were fitted for potential subgroups of the type of hearing loss reported: CHL, MHL, SSD or combinations thereof. Model assumptions were checked by means of normal qq-plot and tests for funnel plot asymmetry and heterogeneity. Tests of heterogeneity were performed using the Cochrane Q statistic and I2 statistic [5], with Q representing the Chi-Square, p the level of evidence and I2 indicating the diversity between studies. If I2< = 25%, studies are regarded homogeneous and if I2> = 75%, high heterogeneity is indicated. Case deletion diagnostics were used to identify potential influential studies. Outcomes are presented in forest plots representing the mean outcomes and confidence intervals (mean [CI]), which are identical to the graphical display in the graph.

## Results

**Fig 1:**
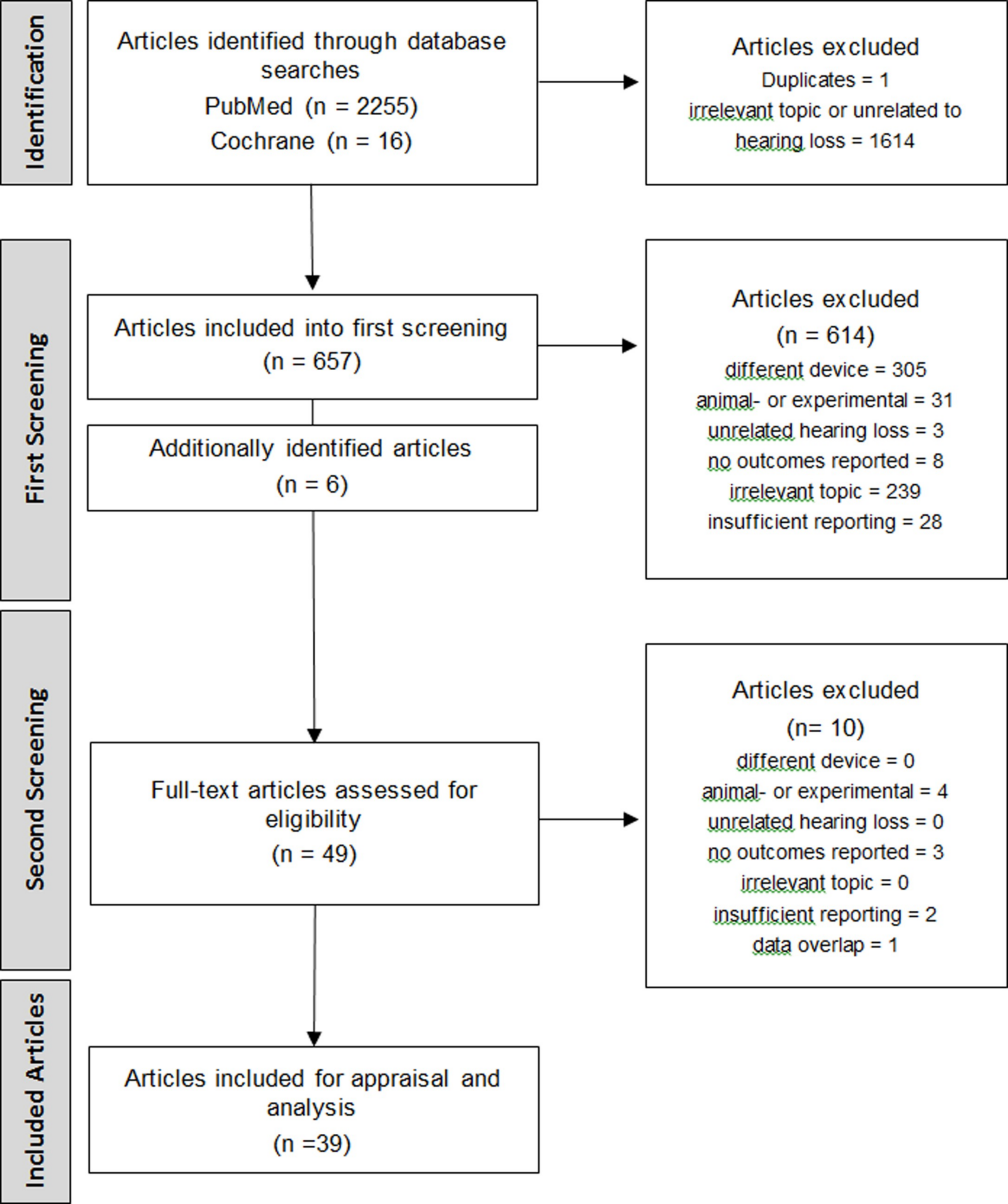
Flow diagram of study selection according to the PRISMA guidelines. (search conducted on Oct 31, 2018).

A total of 2255 records were retrieved through the database searches, and 16 additionally identified citations were included. The title screening revealed 1614 exclusions due to irrelevant topic or the theme being unrelated to treatment or hearing loss itself. The remaining 663 (657 from first-level screening and 6 citations identified through additional bibliography- and systematic review screening) titles and abstracts were screened, unrelated titles were removed (n = 614) (reasons given in Fig 1), and the full texts of the remaining 49 publications were assessed and further articles excluded (Fig 1).

Retrieved publications were subjected to a systematic and thorough screening, selection and validation process (Tables 1 and 3), and outcomes are presented in tables separated by demographic (S1 Table) and surgical information (S1 Table) on the study population, audiological outcomes (S2 Table), subjective outcomes (S3 Table) and safety outcomes with the atBCI (S4 Table).

A total of 39 relevant publications comprising 487 subjects, 303 of whom suffer from conductive hearing loss, 67 from mixed hearing loss and 53 from single-sided deafness were identified for the literature review (for the remaining subjects no details regarding HL were stated). The mean age of the patients in the included studies was 35.6±16.9 years. The youngest implanted subject was 5 years and the oldest candidate was 80 years of age. Due to the size of the floating mass transducer (FMT) of the atBCI, full implantation might require compression of the dura mater or the sigmoid sinus. Information on compression of the sigmoid sinus and/or the dura mater was reported in 10 publications [6–15]. In 39 subjects distributed over 5 studies, sinus compression was reported, and in 8 studies, 49 dura compressions were described; none of them resulted in harmful or further complications for the patient.

**Fig 2:**
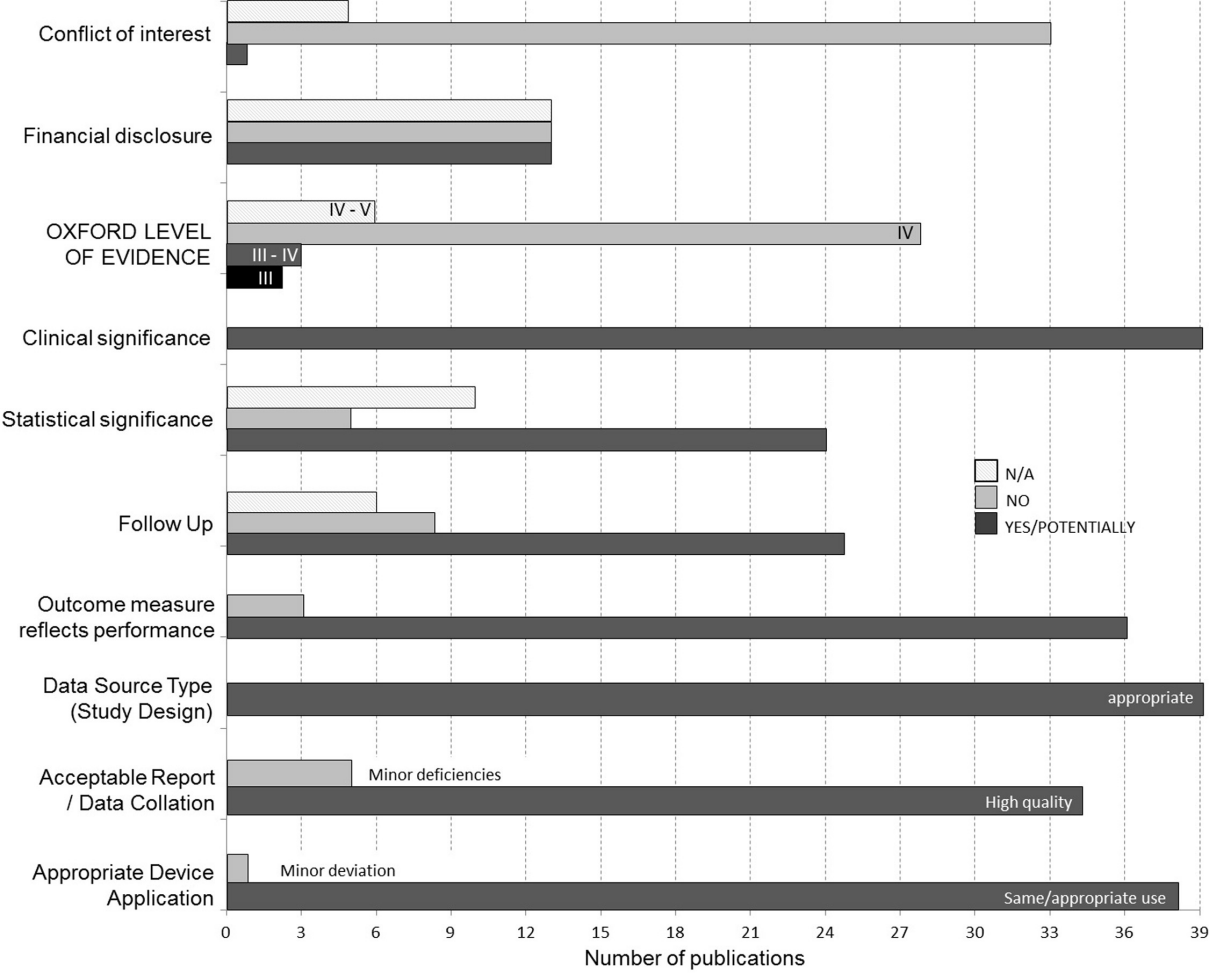
Quality and scientific appraisal of included literature. The graph displays the summary of judgements about each risk of bias domain: N/A not applicable, NO no bias/risk, YES possible bias/risk.

33 studies reported no conflict of interest; one was rated as having a possible conflict of interest and the remaining 5 citations did not report on this matter (N/A). The level of evidence evaluated using the Oxford level of hierarchy system was rated as level IV in two-thirds of the 39 systematically reviewed citations (mainly case series), three publications as a cross between level III and IV and the nine case reports were rated as level IV to V (Fig 2).

Audiological examinations were reported as overall mean outcomes as well as via meta-analysis weighted outcomes for the measure of ‘functional gain’ and ‘word recognition score’. Remaining audiological performance outcomes in noise and subjective outcomes were not reported in a way that a meta-analysis could be performed and therefore overall mean values are stated (S2 Table). Where possible, pediatric outcomes are presented separately from adults. Outcomes are also grouped for conductive and/or mixed as well as SSD indication.

Pure tone hearing thresholds were reported in 29 studies and were in line with the candidacy criteria for the atBCI: mean bone conduction thresholds were all below 45 dB, with no postoperative shift reported.

The functional gain (FG) was measured as the difference between unaided and aided warble tone thresholds, resulting in an overall mean functional gain of 32.7±16dB (S2 Table).

Fourteen articles reporting the functional gain met the inclusion criteria for meta-analysis. The overall FG weighted via meta-analysis exhibited a mean of 30.89 dB SPL [95% 27.53, 34.24](test for heterogeneity: Q = 168.63, df = 18, p<0.001, I2 = 87.9%).

**Fig 3:**
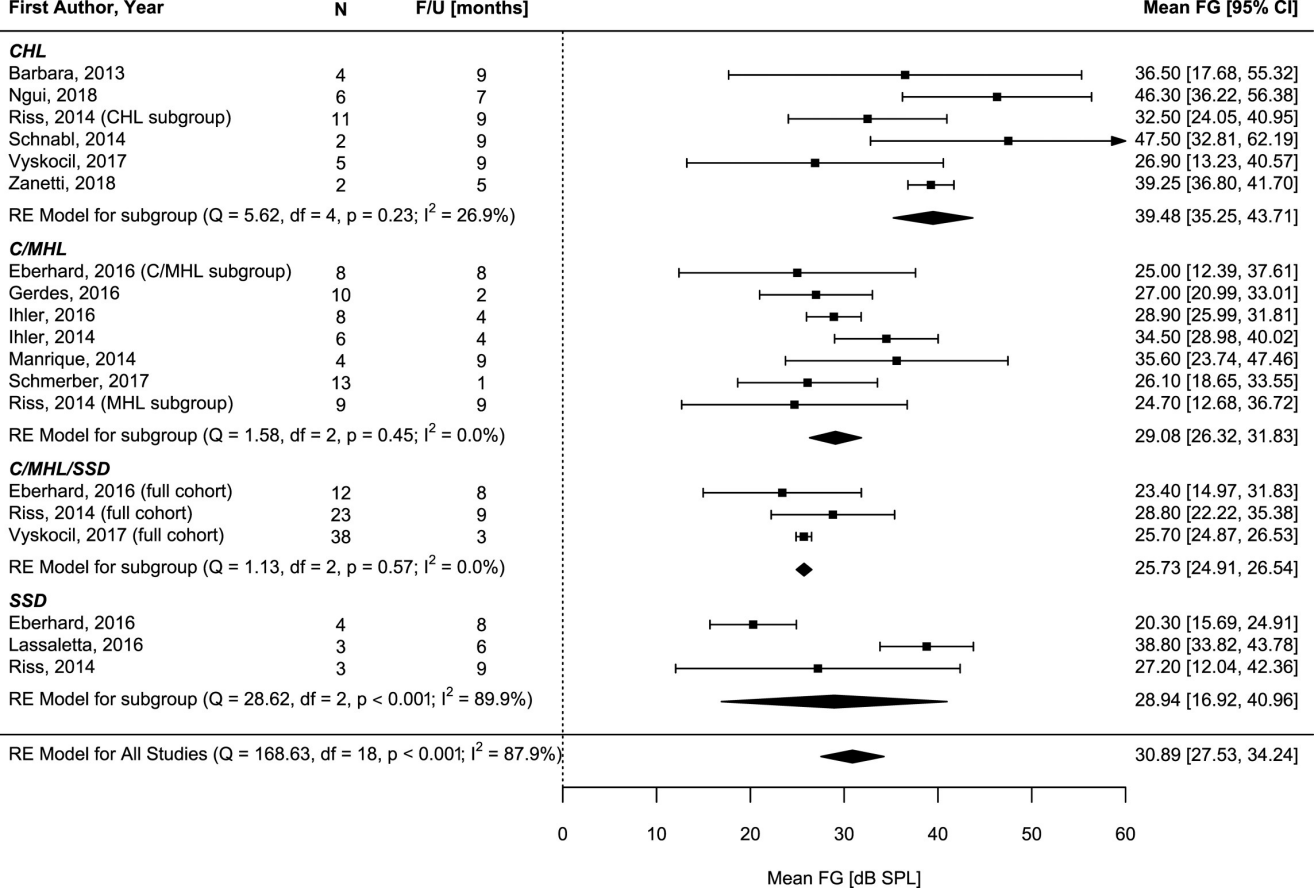
Forest plot of meta-analysis of functional gain outcomes (FG) for patients with conductive hearing loss (CHL), conductive and mixed hearing loss (C/MHL) and single-sided Deafness (SSD).

The meta-analysis for 30 CHL subjects revealed a weighted functional gain of 39.48 dB SPL [95%CI35.25, 43.71](test for heterogeneity: Q = 5.62, df = 4, p = 0.23, I2 = 26.9%)[16–21](Fig 3).

Investigating the mixed hearing loss group (C/MHL), the mean FG resulted in 29.08 dB SPL [26.32, 31.83](test for heterogeneity: Q = 1.58, df = 2, p = 0.45, I2 = 0.0%)(n = 58)[7, 22–25, 18, 13].

In the outcomes reporting on 10 subjects with SSD, the average weighted functional gain was 28.94 dB SPL [16.92, 40.96](test for heterogeneity: Q = 28.62, df = 2, p<0.001, I2 = 89.9%) [7, 10, 18] (Fig 3).

Speech tests used included the Italian bisyllables, Freiburger monosyllables, Mainzer monosyllables, Göttinger Kindersprachtest, Dantale (Danish), Oldenburg sentence test, dissyllabic Fournier and Spanish bisyllables words and numbers lists. The preoperative and postoperative results can be seen in the S2 Table. Speech understanding in quiet was assessed in 27 publications [16, 26, 27, 7, 28, 22, 29, 24, 30, 10, 31, 25, 32–34, 18, 12, 13, 35, 36, 20, 14, 37, 38, 21, 39], resulting in a mean unaided WRS score of 25.73±23.64% improving to 84.48±15.09% in the aided condition, resulting in an overall mean improvement of almost 60% (S2 Table).

**Fig 4:**
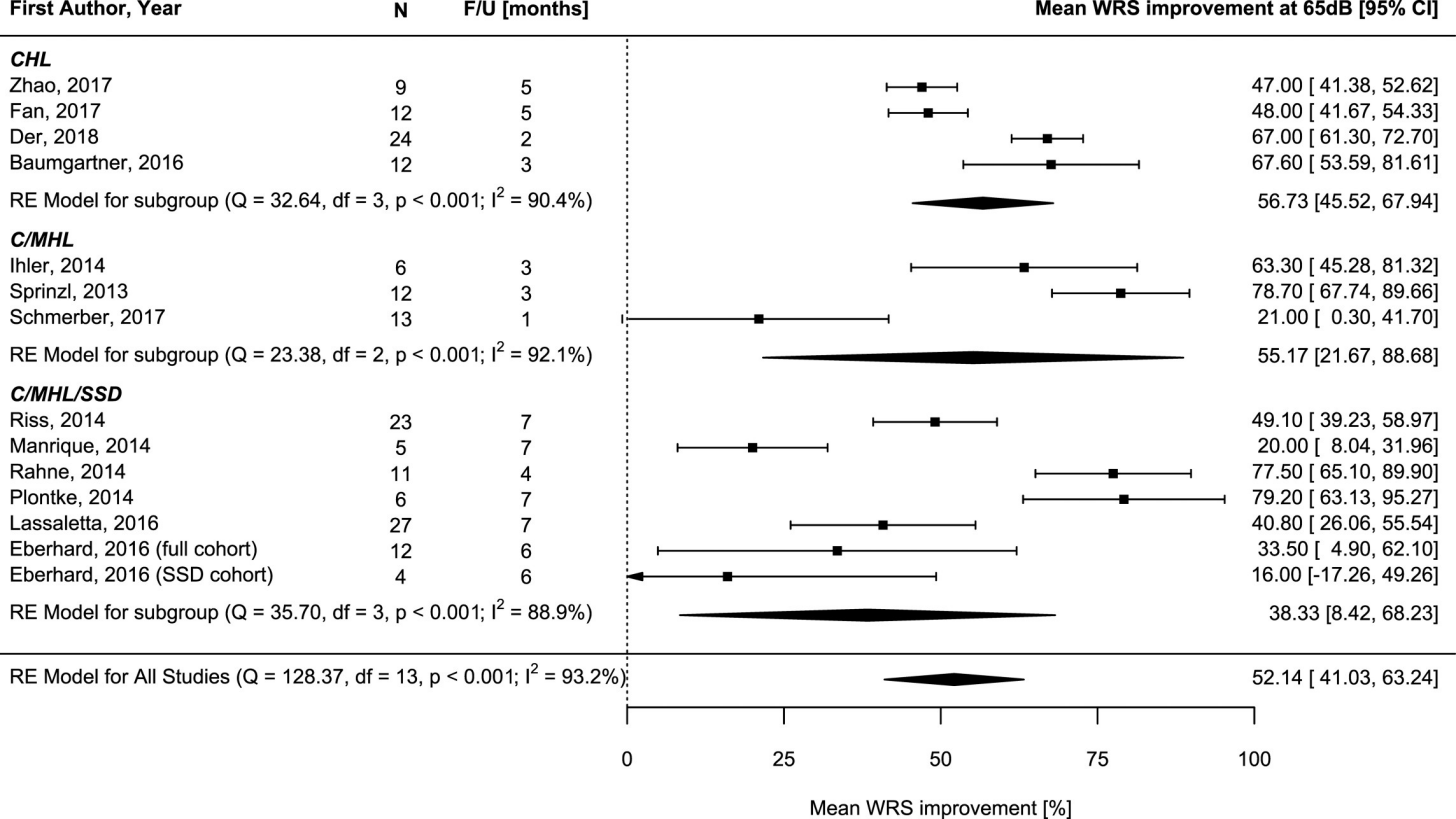
Forest plot of meta-analysis of improvement in word recognition score (WRS) at 65dB.

The meta-analysis outcomes for reported mean word recognition scores including SD at 65 dB SPL in the CHL group resulted in an improvement of 56.73% [95%CI 45.52, 67.94](test for heterogeneity: Q = 32.64, df = 3, p<0.001, I2 = 90.4%)(n = 57)(F/U range: 2–5 months) (Fig 4) [26, 6, 28, 39]. Outcomes were similar in the C/MHL group, reported in 3 studies comprising 31 subjects (mean WRS improvement 55.14%)[21.67, 88.68](test for heterogeneity: Q = 23.38, df = 2, p<0.001, I2 = 92.1%)(F/U ranged between 1 and 3 months)[24, 13, 35]. Subjects with assigned hearing loss (C/MHL/SSD) were reported in seven studies with 78 subjects and revealed a mean improvement of 38.33% [95%CI 8.42, 63.24](test for heterogeneity: Q = 35.7, df = 3, p<0.001, I2 = 88.9%), which is low due to the SSD subjects within this cohort reporting a WRS of 16% [95%CI -17.26, 49.26](Fig 4)[7, 10, 25, 32, 33, 18].

Speech in noise tests used included the QuickSIN and BKB-SIN with four-talker babble noise, Italian bisyllables, Freiburger monosyllables, Dantale II (Danish), Oldenburg sentence test, words and numbers lists. The preoperative and postoperative results reported can be seen in the S2 Table.

Results for speech in noise tests could not be compared directly in a meta-analysis due to the individual test set-up and inconsistency in reporting for the different studies. However, an improvement in speech understanding in noise was observed with the atBCI in all studies for subjects with CHL or MHL (S2 Table) [23, 8, 13, 20, 38, 21].

Mean aided signal-to-noise ratio (SNR) values in a total of 54 individuals with CHL and MHL were found to range from +2.9 dB to -6.1 dB SNR, compared to + 11.5 to -3.8 SNR unaided [7, 40, 41, 33, 42, 43]. Studies that investigated a setting in which speech was presented from a loudspeaker in front of the patient reported an average improvement in SNR of 5.5 dB [7, 41, 33, 42, 43]. Looking at individual data, large variability was observed between individuals, but outcomes were always in favour of the atBCI-aided condition.

For single-sided deaf subjects, an improvement in speech understanding in noise was observed with the atBCI [9, 10, 12, 13]. This improvement was especially seen when noise was presented from the normal-hearing side and speech was provided on the deaf side.

Sound localization ability is usually investigated with white noise presented at a level of 65 dB SPL from certain, usually randomized angles (α) of -90°, -45°, 0°, 45°, or 90°. The ability to localize sound is then calculated as the angle detection error (ADE,°), which is the difference, Δαi, between the actual angle and the detected angle [32, 33, 38]. Sound localization performance can also be quantified using the root mean square (RMS) error [20]. The effect on auditory localization was assessed in four studies in 40 patients with C/MHL or SSD. Weiss et al. and Rahne et al. found no significant difference between the unaided and atBCI conditions for auditory localization in the horizontal plane in 18 subjects and 11 subjects, respectively [33, 38]. Vyskocil et al. found in five users that the atBCI implant improved sound localization significantly and that the benefit concerning sound source localization depended on the location of the sound source [20]. Plontke et al. found an improvement from 60.3±36.4 in the unaided condition to 36±34.5 in the atBCI aided condition [32]. Currently, numbers are too small to draw conclusions or perform a meta-analysis on the benefit on sound localization in the aided situation.

Sixteen publications with 215 subjects reported on subjective outcomes after atBC implantation (S3 Table) [27, 7, 28, 22–24, 8–10, 44, 17, 12, 13, 35, 38, 21].

The Abbreviated Profile of Hearing Aid Benefit (APHAB) was the most frequently used questionnaire and was administered in seven publications evaluating 71 subjects [28, 22, 23, 44, 12, 13, 21]. Patients with CHL and MHL reported a significant benefit from the atBCI in the APHAB subscale score for ease of communication (scores ranged from 8 to 10). The other subscales included background noise (scores ranged from 12 to 21), reverberation (scores ranged from 1 to 25) and aversiveness (scores ranged from 30 to 52) [28, 23, 44, 13, 21]. In two studies [40, 41] this was statistically significant. The subscale aversiveness was rated worse in the aided condition in all three studies.

The Glasgow Benefit Inventory (GBI) was reported in five studies in 47 subjects [27, 23, 24, 44, 13]. The total GBI score was positive for all users, reflecting subjective improvement of well-being since implantation of the active transcutaneous bone conduction device, with outcomes ranging from 30 to 39 in subjects with CHL and MHL. The International Outcome Inventory for Hearing Aids (IOI-HA) was administered in two studies for 20 patients with CHL and MHL and 4 with SSD [7, 45]; the mean score was between 4 and 5 on all items. The PEACH, SSQ and BBSS questionnaire were each used once in subjects with CHL and MHL. Patients were satisfied with the device, stating improvement in their quality of life. The HDSS (Hearing Device Satisfaction Scale) was used in three studies (n = 30) [26, 17, 35]. Subjective device satisfaction ranged from 49 to 100% with a mean of 98%. The SSQ (Speech, Spatial and Qualities of Hearing) questionnaire was applied in two studies with 8 participants [7, 21].

A modified BBSS (Bern Benefit in Single-Sided Deafness) questionnaire was also used in two studies to evaluate the subjective outcome in conductive and mixed hearing loss patients (n = 18)[12, 43]. On a scale of -5 to +5, the average was between 2.7 and 2.8 on all questions answered by the 27 investigated subjects. Subjective measurements were also performed in subjects with SSD using the APHAB, the BBSS, the GBI and the IOI-HA, evaluating the impact of the active transcutaneous bone conduction implant on their quality of life [9, 10, 13]. The BBSS reported an average benefit of 2.8 compared to the unaided condition. The total score on the GBI was approximately 15 and the IOI-HA showed the atBCI to have a benefit over usage of a conventional hearing aid. The follow-up period of investigation spanned 11 to 25 months. Further reported outcomes of the different questionnaires can be seen in the S3 Table.

Outcomes in children (subjects 18 years or younger) were reported in six publications. For children with CHL or MHL the average functional gain was 34 dB for 77 implantations [6, 28, 24, 10, 33, 20, 14]. Also, children reached an average aided sound field threshold close to normal hearing with the atBCI, i.e. 24 dB HL for 67 implants [6, 28, 8].

Baumgartner et al. reported a significant improvement in warble tone thresholds from preoperative testing to 3-month postoperative testing (all frequencies)[26]. Preoperative mean monosyllabic WRS was 14.5% (SD 21.6) and increased at 1 month after implantation to 67.2% (SD 17.9) and to 82.1% (SD 12.1) after 3 months. The preoperative SRT50 was 72.7 (SD 5.9) dB SPL and improved 1 month after surgery to 52.5 (8.2) dB SPL and after 3 months to 45.2 (6.9) dB SPL. Furthermore, no significant differences in bone conduction thresholds between preoperative testing and 3-month postoperative testing were noted in the results of the paired sample t test, suggesting that the intervention did not affect the children’s residual hearing. Riss et al. reported on six pediatric patients (6 to 17 years of age) suffering from atresia [18]. There was no separate evaluation of the pediatric data or single subjects, but patients with atresia (n = 11) had an average functional hearing gain of 32.5 dB (± 14.3 SD) with the atBCI.

Vyskocil et al. reported on two pediatric patients (14 and 17 years old) suffering from conductive hearing loss due to microtia and atresia [20]. Mean WRS at 65 dB improved from 2.5% unaided to 47.5% in the aided condition. Mean speech reception threshold in the S0N0 setting improved by 10.2 dB and by 9.5 dB in the S-90N90 setting. The root mean square angle error decreased in both users with a median change of 4.1 degrees.

Subjective outcomes in children were reported by Baumgartner et al., investigating the Hearing Device Satisfaction Scale (HDSS) in children aged 5 to 17 years (n = 12) [26]. Outcomes ranged from 55 to 100% (mean 88%). The average length of device use was 11.2 hours per day. One subject reported only moderate satisfaction, despite good audiological outcomes. The patient described experiencing an unfamiliar hearing sensation with the device, and the AP was re-fitted according to the patient’s needs and the patient subsequently reported a higher level of satisfaction.

Safety of the device was assessed by collecting information on complications during surgery and adverse events in the postoperative period (S4 and S5 Tables). Twenty-five [16, 26, 27, 6, 7, 29, 24, 30, 8–10, 31, 11, 25, 17, 33, 18, 13, 35, 36, 14, 38, 15, 21, 46] publications out of the 39 identified citations reported on complications and adverse events, out of which ten citations (n = 259; 90.6%), explicitly stated that no complications occurred during the full study period [16, 27, 30, 10, 31, 11, 25, 38, 15, 21]. A total of 286 ears were evaluated for safety outcomes over a mean follow-up period of 11.7±4.5 months (range: 3–36 months). The reported complications were categorized into minor and major complications, with a major complication described as requiring surgical attention leading to revision surgery or explantation (S5 Table).

**Fig 5:**
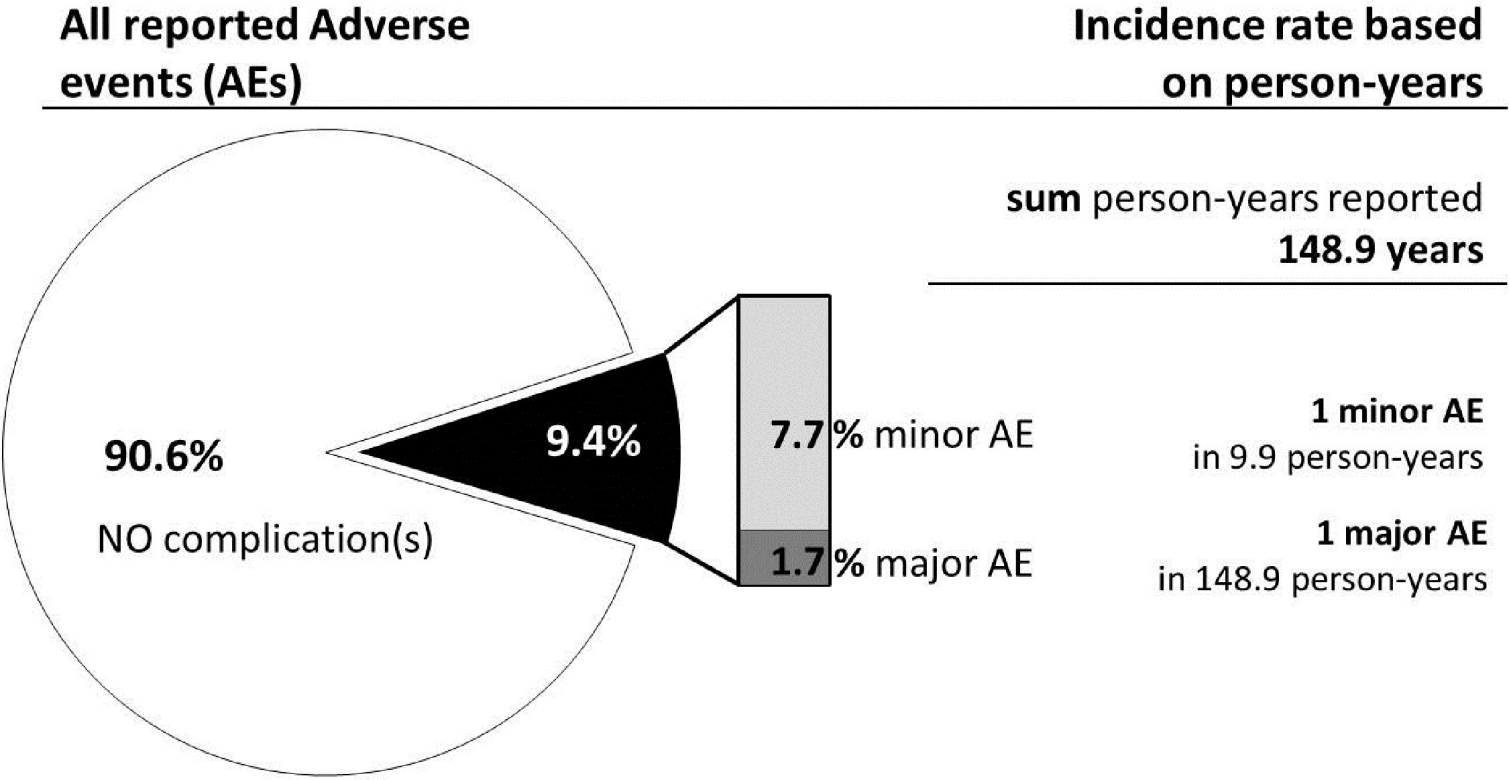
Safety outcomes (separated into all reported outcomes (left) and AEs with reported follow up-time (F/U)(right).

Out of 286 ears under investigation, 259 reported no complications (90.6%). Minor complications in 22 ears resulted in a 7.7% rate over a cumulative period of reported mean follow-up of 12.7 years (mean: 11.7 months ± 4.5) [26, 6, 7, 29, 24, 8, 9, 17, 13, 35, 36, 14, 46]. Major complications occurred in 3 studies comprising 5 ears, which equates to 1.7%. Details can be seen in Fig 5, and the S4 and S5 Tables. The persons-years, the actual time-at-risk in years per person, could be retrieved from a total of 13 studies and summed up to 148.9. The resulting incidence rate or person-time rate can be summarized as 7 major adverse events (AE) in 1000 subjects per follow-up year and as 1 in 10 minor AEs per year of follow-up (Fig 5).

## Discussion

Substantial and stable benefit for patients with C/MHL and SSD who underwent active transcutaneous bone conduction device implantation was shown in 39 citations. Benefit was defined in terms of hearing thresholds and speech recognition in quiet, as well as speech discrimination and functional gain. Averaging the studies reporting all indication groups, a weighted functional gain of 31 dB could be achieved (C/MHL/SSD). A mean weighted benefit in word recognition score at 65dB of 52.1% was found with the atBCI for all subject groups (C/MHL/SSD), with the SSD group still performing well but not as high as the other hearing loss types (38.3% WRS improvement). The conductive hearing loss group benefitted the most, with a score of 56.7%. Speech understanding in noise also improved significantly. The comparison with other, non-transcutaneous and non-active devices has shown similar results in quiet and noise with a ceiling effect, although their functional gains are different in the low and high frequencies [45].

The device’s transcutaneous technology avoids several complications found in percutaneous bone conduction implants including skin reaction, growth of skin over the abutment, implant extrusion, and wound infection [47, 44, 34, 46]. The complication rate reported for atBCI recipients was considerably lower with one minor event in 9.9 person-years, compared to other devices, especially percutaneous bone-anchored hearing aids such as the BAHA (Cochlear Limited, Australia)[48]. The low overall incidence rate with the atBCI, namely the Bonebridge is also reflected in the fact that the rate of major adverse events has been remarkably low, with one major incidence in 148.9 person-years.

The currently reviewed device is the only available active transcutaneous system. Other active bone conduction devices that utilize a percutaneous screw still have to battle high, especially skin-related complication rates. This led to the development of bone-anchored systems that use transcutaneous magnetic coupling to enable sound conduction (screw-based transcutaneous device). Despite being a closed-skin option that aims to avoid skin complications, reports of skin irritations, like edema and erythema and even necrosis seem to be a common occurrence [48–61]. Implantation of both types of devices, the active transcutaneous system as well as the active screw-based system, is relatively simple and quick [1] but the latter requires osseointegration before activation. Both device surgeries may be performed under local anaesthesia as the reported surgical time for the atBCI ranges between 30 and 90 minutes [1, 6, 21, 26, 40] and for the screw-based transcutaneous device between 30 and 82 minutes [62–66].

Nonetheless, the limitations of this systematic review need to be emphasized in regard to the reporting standard, especially in outcomes such as word recognition scores in quiet and in noise. Test apparatus and language varies across study sites from various countries and may impede outcome comparisons. Furthermore, the Level of Evidence of the reviewed literature, comprising 2a/3a studies (cohort studies and case-control studies) reduces the strength of outcome variables. For this reason, a meta-analysis, was performed to overcome this, when possible. In addition, no other active transcutaneous bone conduction hearing implant is available at the moment and therefore no comparative evidence could be extracted. Last but not least, long term outcomes remain unknown and must be further investigated to check the maintenance of functional gain / WRS as well as potential future long-term complications.

The atBCI is very well tolerated up to 36 months following implantation, improves audiometric thresholds and intelligibility for speech in quiet and noise, and gives satisfaction to patients with mixed and conductive hearing loss as well as those with SSD. The improved audiological benefit with the active transcutaneous bone conduction implant reviewed here is furthermore reflected in the high levels of subjective satisfaction reported by users via several questionnaires (e.g. APHAB, GBI, HISQUI etc.) and the remarkably low complication rate.

To extend the evaluation of the atBCI, more data and an even longer follow-up is required.

## Conclusion

The only active transcutaneous bone conduction technology presented here avoids several well-known complications of the percutaneous bone conduction implants. The complication rate reported was low, with one minor event in 9.9 person-years. The low overall incidence rate with the atBCI is also reflected in the ‘severity of events’, with the remarkably low major incidence rate of one in 148.9 person-years. Based on the reviewed outcomes it can furthermore be concluded that the Bonebridge is an effective solution for adults and children suffering from conductive and/or mixed hearing loss as well as single-sided deaf subjects, with the advantage of an intact skin condition after implantation.

Based on the audiological outcomes, high patient satisfaction and low complication rates, the authors conclude that this active transcutaneous bone conduction implant is a safe and effective treatment for patients suffering from hearing loss within the device’s indication criteria.

## Supporting information

S1 Table Demographic and surgical information on the study population CHL conductive hearing loss, MHL mixed hearing loss, SSD single-sided deafness, atBCI active transcutaneous bone conduction device, N/A information not available, ± standard deviation, M male, F female, COM chronic otitis media. (DOCX)

S2 Table Audiological outcomes with the atBCI AC air conduction, BC bone conduction, N/A not available/not reported, SPL speech presentation level, ± standard deviation, SNR signal to noise ratio, PTA4 (pure tone average over freq. 0.5, 1, 2, 4kHz), WRS word recognition score, BCHA bone conduction hearing aid, SRT speech reception threshold, SDS speech discrimination score, # data extracted from figure. (DOCX)

S3 Table Subjective outcomes with the active transcutaneous bone conduction implant (atBCI) # data extracted from figure. (DOCX)

S4 Table Safety outcomes (F/U follow up, # number of). (DOCX)

S5 Table Safety outcome categories (AE adverse event, RS revision surgery; expl. explantation, # number of). (DOCX)

S1 Checklist PRISMA 2009 checklist. (DOC)

## References

[1] Sprinzl G.M., Wolf-Magele A. The Bonebridge Bone Conduction Hearing Implant: indication criteria, surgery and a systematic review of the literature. Clinical otolaryngology: official journal of ENT-UK; official journal of Netherlands Society for Oto-Rhino-Laryngology & Cervico-Facial Surgery. 2016;41(2):131–43. 10.1111/coa.12484 26073720

[2] R Core Team. R: A language and environment for statistical computing.: R Foundation for Statistical Computing, Vienna, Austria; 2016 [Available from: https://www.R-project.org/.

[3] RStudio Team. RStudio: Integrated Development for R. RStudio: RStudio, Inc., Boston, MA; 2015 [Available from: http://www.rstudio.com/.

[4] Viechtbauer W. Conducting Meta-Analyses in R with the metafor Package. Journal of Statistical Software. 2010;36(3):48.

[5] Higgins J.P., Thompson S.G., Deeks J.J., Altman D.G. Measuring inconsistency in meta-analyses. BMJ. 2003;327(7414):557–60. 10.1136/bmj.327.7414.557 12958120 PMC192859

[6] Der C., Bravo-Torres S., Pons N. Active Transcutaneous Bone Conduction Implant: Middle Fossa Placement Technique in Children With Bilateral Microtia and External Auditory Canal Atresia. Otology & neurotology: official publication of the American Otological Society, American Neurotology Society and European Academy of Otology and Neurotology. 2018;39(5):e342–e8. 10.1097/MAO.0000000000001809 29664868

[7] Eberhard K.E., Olsen S.O., Miyazaki H., Bille M., Caye-Thomasen P. Objective and Subjective Outcome of a New Transcutaneous Bone Conduction Hearing Device: Half-year Follow-up of the First 12 Nordic Implantations. Otology & neurotology: official publication of the American Otological Society, American Neurotology Society and European Academy of Otology and Neurotology. 2016;37(3):267–75. 10.1097/MAO.0000000000000969 26859460

[8] Kulasegarah J., Burgess H., Neeff M., Brown C.R.S. Comparing audiological outcomes between the Bonebridge and bone conduction hearing aid on a hard test band: Our experience in children with atresia and microtia. International journal of pediatric otorhinolaryngology. 2018;107:176–82. 10.1016/j.ijporl.2018.01.032 29501302

[9] Laske R.D., Roosli C., Pfiffner F., Veraguth D., Huber A.M. Functional Results and Subjective Benefit of a Transcutaneous Bone Conduction Device in Patients With Single-Sided Deafness. Otology & neurotology: official publication of the American Otological Society, American Neurotology Society and European Academy of Otology and Neurotology. 2015;36(7):1151–6. 10.1097/MAO.0000000000000791 26111077

[10] Lassaletta L., Calvino M., Zernotti M., Gavilan J. Postoperative pain in patients undergoing a transcutaneous active bone conduction implant (Bonebridge). European archives of oto-rhino-laryngology: official journal of the European Federation of Oto-Rhino-Laryngological Societies (EUFOS): affiliated with the German Society for Oto-Rhino-Laryngology—Head and Neck Surgery. 2016;273(12):4103–10. 10.1007/s00405-016-3972-y 26968179

[11] Law E.K., Bhatia K.S., Tsang W.S., Tong M.C., Shi L. CT pre-operative planning of a new semi-implantable bone conduction hearing device. European radiology. 2016;26(6):1686–95. 10.1007/s00330-015-3983-x 26385806

[12] Salcher R., Zimmermann D., Giere T., Lenarz T., Maier H. Audiological Results in SSD With an Active Transcutaneous Bone Conduction Implant at a Retrosigmoidal Position. Otology & neurotology: official publication of the American Otological Society, American Neurotology Society and European Academy of Otology and Neurotology. 2017;38(5):642–7. 10.1097/MAO.0000000000001394 28375939

[13] Schmerber S., Deguine O., Marx M., Van de Heyning P., Sterkers O., Mosnier I., et al Safety and effectiveness of the Bonebridge transcutaneous active direct-drive bone-conduction hearing implant at 1-year device use. European archives of oto-rhino-laryngology: official journal of the European Federation of Oto-Rhino-Laryngological Societies (EUFOS): affiliated with the German Society for Oto-Rhino-Laryngology—Head and Neck Surgery. 2017;274(4):1835–51. 10.1007/s00405-016-4228-6 27475796

[14] Vyskocil E., Riss D., Arnoldner C., Hamzavi J.S., Liepins R., Kaider A., et al Dura and sinus compression with a transcutaneous bone conduction device—hearing outcomes and safety in 38 patients. Clinical otolaryngology: official journal of ENT-UK; official journal of Netherlands Society for Oto-Rhino-Laryngology & Cervico-Facial Surgery. 2017;42(5):1033–8. 10.1111/coa.12793 27860393

[15] Wimmer W., Gerber N., Guignard J., Dubach P., Kompis M., Weber S., et al Topographic bone thickness maps for Bonebridge implantations. European archives of oto-rhino-laryngology: official journal of the European Federation of Oto-Rhino-Laryngological Societies (EUFOS): affiliated with the German Society for Oto-Rhino-Laryngology—Head and Neck Surgery. 2015;272(7):1651–8. 10.1007/s00405-014-2976-8 24627076

[16] Barbara M., Perotti M., Gioia B., Volpini L., Monini S. Transcutaneous bone-conduction hearing device: audiological and surgical aspects in a first series of patients with mixed hearing loss. Acta oto-laryngologica. 2013;133(10):1058–64. 10.3109/00016489.2013.799293 23768011

[17] Ngui L.X., Tang I.P. Bonebridge transcutaneous bone conduction implant in children with congenital aural atresia: surgical and audiological outcomes. The Journal of laryngology and otology. 2018;132(8):693–7. 10.1017/S0022215118001123 30008276

[18] Riss D., Arnoldner C., Baumgartner W.D., Blineder M., Flak S., Bachner A., et al Indication criteria and outcomes with the Bonebridge transcutaneous bone-conduction implant. The Laryngoscope. 2014;124(12):2802–6. 10.1002/lary.24832 25142577

[19] Schnabl J., Wolf-Magele A., Pok S.M., Schoerg P., Hirtler L., Schloegel M., et al Intraoperative measurement for a new transcutaneous bone conduction hearing implant. Otology & neurotology: official publication of the American Otological Society, American Neurotology Society and European Academy of Otology and Neurotology. 2014;35(7):1242–7. 10.1097/MAO.0000000000000351 24751748

[20] Vyskocil E., Liepins R., Kaider A., Blineder M., Hamzavi S. Sound Localization in Patients With Congenital Unilateral Conductive Hearing Loss With a Transcutaneous Bone Conduction Implant. Otology & neurotology: official publication of the American Otological Society, American Neurotology Society and European Academy of Otology and Neurotology. 2017;38(3):318–24. 10.1097/MAO.0000000000001328 28079678

[21] Zanetti D., Di Berardino F. A Bone Conduction Implantable Device as a Functional Treatment Option in Unilateral Microtia with Bilateral Stapes Ankylosis: A Report of Two Cases. The American journal of case reports. 2018;19:82–9. 10.12659/AJCR.904907 29358571 PMC5789751

[22] Gerdes T., Salcher R., Schwab B., Lenarz T., Maier H. Comparison of Audiological Results Between a Transcutaneous and a Percutaneous Bone Conduction Instrument in Conductive Hearing Loss. Otology & neurotology: official publication of the American Otological Society, American Neurotology Society and European Academy of Otology and Neurotology. 2016. 10.1097/MAO.0000000000001010 27093021

[23] Ihler F., Blum J., Berger M.U., Weiss B.G., Welz C., Canis M. The Prediction of Speech Recognition in Noise With a Semi-Implantable Bone Conduction Hearing System by External Bone Conduction Stimulation With Headband: A Prospective Study. Trends in hearing. 2016;20. 10.1177/2331216516669330 PMC5051673 27698259

[24] Ihler F., Volbers L., Blum J., Matthias C., Canis M. Preliminary functional results and quality of life after implantation of a new bone conduction hearing device in patients with conductive and mixed hearing loss. Otology & neurotology: official publication of the American Otological Society, American Neurotology Society and European Academy of Otology and Neurotology. 2014;35(2):211–5. 10.1097/MAO.0000000000000208 24448279

[25] Manrique M., Sanhueza I., Manrique R., de Abajo J. A new bone conduction implant: surgical technique and results. Otology & neurotology: official publication of the American Otological Society, American Neurotology Society and European Academy of Otology and Neurotology. 2014;35(2):216–20. 10.1097/MAO.0000000000000253 24448280

[26] Baumgartner W.D., Hamzavi J.S., Boheim K., Wolf-Magele A., Schlogel M., Riechelmann H., et al A New Transcutaneous Bone Conduction Hearing Implant: Short-term Safety and Efficacy in Children. Otology & neurotology: official publication of the American Otological Society, American Neurotology Society and European Academy of Otology and Neurotology. 2016;37(6):713–20. 10.1097/MAO.0000000000001038 27153327

[27] Bianchin G., Bonali M., Russo M., Tribi L. Active bone conduction system: outcomes with the Bonebridge transcutaneous device. ORL; journal for oto-rhino-laryngology and its related specialties. 2015;77(1):17–26. 10.1159/000371425 25661010

[28] Fan X., Wang Y., Wang P., Fan Y., Chen Y., Zhu Y., et al Aesthetic and hearing rehabilitation in patients with bilateral microtia-atresia. International journal of pediatric otorhinolaryngology. 2017;101:150–7. 10.1016/j.ijporl.2017.08.008 28964287

[29] Hassepass F., Bulla S., Aschendorff A., Maier W., Traser L., Steinmetz C., et al The bonebridge as a transcutaneous bone conduction hearing system: preliminary surgical and audiological results in children and adolescents. European archives of oto-rhino-laryngology: official journal of the European Federation of Oto-Rhino-Laryngological Societies (EUFOS): affiliated with the German Society for Oto-Rhino-Laryngology—Head and Neck Surgery. 2015;272(9):2235–41. 10.1007/s00405-014-3137-9 24970289

[30] Kim M. Bonebridge Implantation for Conductive Hearing Loss in a Patient with Oval Window Atresia. The journal of international advanced otology. 2015;11(2):163–6. 10.5152/iao.2015.1341 26381009

[31] Lassaletta L., Sanchez-Cuadrado I., Munoz E., Gavilan J. Retrosigmoid implantation of an active bone conduction stimulator in a patient with chronic otitis media. Auris, nasus, larynx. 2014;41(1):84–7. 10.1016/j.anl.2013.04.004 23722197

[32] Plontke S.K., Radetzki F., Seiwerth I., Herzog M., Brandt S., Delank K.S., et al Individual computer-assisted 3D planning for surgical placement of a new bone conduction hearing device. Otology & neurotology: official publication of the American Otological Society, American Neurotology Society and European Academy of Otology and Neurotology. 2014;35(7):1251–7. 10.1097/MAO.0000000000000405 24770405

[33] Rahne T., Seiwerth I., Gotze G., Heider C., Radetzki F., Herzog M., et al Functional results after Bonebridge implantation in adults and children with conductive and mixed hearing loss. European archives of oto-rhino-laryngology: official journal of the European Federation of Oto-Rhino-Laryngological Societies (EUFOS): affiliated with the German Society for Oto-Rhino-Laryngology—Head and Neck Surgery. 2015;272(11):3263–9. 10.1007/s00405-014-3403-x 25425039

[34] Rainsbury J.W., Williams B.A., Gulliver M., Morris D.P. Preoperative headband assessment for semi-implantable bone conduction hearing devices in conductive hearing loss: is it useful or misleading? Otology & neurotology: official publication of the American Otological Society, American Neurotology Society and European Academy of Otology and Neurotology. 2015;36(2):e58–62. 10.1097/MAO.0000000000000695 25548890

[35] Sprinzl G., Lenarz T., Ernst A., Hagen R., Wolf-Magele A., Mojallal H., et al First European multicenter results with a new transcutaneous bone conduction hearing implant system: short-term safety and efficacy. Otology & neurotology: official publication of the American Otological Society, American Neurotology Society and European Academy of Otology and Neurotology. 2013;34(6):1076–83. 10.1097/MAO.0b013e31828bb541 23714710

[36] Tsang W.S., Yu J.K., Bhatia K.S., Wong T.K., Tong M.C. The Bonebridge semi-implantable bone conduction hearing device: experience in an Asian patient. The Journal of laryngology and otology. 2013;127(12):1214–21. 10.1017/S0022215113002144 24168962

[37] Weiss B.G., Bertlich M., Scheele R., Canis M., Jakob M., Sohns J.M., et al Systematic radiographic evaluation of three potential implantation sites for a semi-implantable bone conduction device in 52 patients after previous mastoid surgery. European archives of oto-rhino-laryngology: official journal of the European Federation of Oto-Rhino-Laryngological Societies (EUFOS): affiliated with the German Society for Oto-Rhino-Laryngology—Head and Neck Surgery. 2017;274(8):3001–9. 10.1007/s00405-017-4609-5 28528370

[38] Weiss R., Leinung M., Baumann U., Weissgerber T., Rader T., Stover T. Improvement of speech perception in quiet and in noise without decreasing localization abilities with the bone conduction device Bonebridge. European archives of oto-rhino-laryngology: official journal of the European Federation of Oto-Rhino-Laryngological Societies (EUFOS): affiliated with the German Society for Oto-Rhino-Laryngology—Head and Neck Surgery. 2017;274(5):2107–15. 10.1007/s00405-016-4434-2 28032241

[39] Zhao S., Gong S., Han D., Zhang H., Ma X., Li Y., et al Round window application of an active middle ear implant (AMEI) system in congenital oval window atresia. Acta oto-laryngologica. 2016;136(1):23–33. 10.3109/00016489.2014.1003091 26493073

[40] Gerdes T., Salcher R.B., Schwab B., Lenarz T., Maier H. Comparison of Audiological Results Between a Transcutaneous and a Percutaneous Bone Conduction Instrument in Conductive Hearing Loss. Otology & neurotology: official publication of the American Otological Society, American Neurotology Society and European Academy of Otology and Neurotology. 2016;37(6):685–91. 10.1097/MAO.0000000000001010 27093021

[41] Ihler F., Blum J., Berger M.-U., Weiss B.G., Welz C., Canis M. The Prediction of Speech Recognition in Noise With a Semi-Implantable Bone Conduction Hearing System by External Bone Conduction Stimulation With Headband: A Prospective Study. Trends in Hearing. 2016;20:2331216516669330. 10.1177/2331216516669330 PMC5051673 27698259

[42] Vyskocil E., Liepins R., Kaider A., Blineder M., Hamzavi S. Sound Localization in Patients With Congential Unilateral Conductive Hearing Loss With a Transcutaneous Bone Conduction Implant. Otology & neurotology: official publication of the American Otological Society, American Neurotology Society and European Academy of Otology and Neurotology. 2017. 10.1097/MAO.0000000000001328 28079678

[43] Weiss R., Leinung M., Baumann U., Weißgerber T., Rader T., Stöver T. Improvement of speech perception in quiet and in noise without decreasing localization abilities with the bone conduction device Bonebridge. European Archives of Oto-Rhino-Laryngology. 2016:1–9. 10.1007/s00405-016-4434-2 28032241

[44] Monini S., Bianchi A., Talamonti R., Atturo F., Filippi C., Barbara M. Patient satisfaction after auditory implant surgery: ten-year experience from a single implanting unit center. Acta oto-laryngologica. 2017;137(4):389–97. 10.1080/00016489.2016.1258733 27918233

[45] Schmerber S., Deguine O., Marx M., Van de Heyning P., Sterkers O., Mosnier I., et al Safety and effectiveness of the Bonebridge transcutaneous active direct-drive bone-conduction hearing implant at 1-year device use. European archives of oto-rhino-laryngology: official journal of the European Federation of Oto-Rhino-Laryngological Societies (EUFOS): affiliated with the German Society for Oto-Rhino-Laryngology—Head and Neck Surgery. 2016. 10.1007/s00405-016-4228-6 27475796

[46] Zernotti M.E., Di Gregorio M.F., Galeazzi P., Tabernero P. Comparative outcomes of active and passive hearing devices by transcutaneous bone conduction. Acta oto-laryngologica. 2016;136(6):556–8. 10.3109/00016489.2016.1143119 26981711

[47] Jovankovicova A., Stanik R., Kunzo S., Majakova L., Profant M. Surgery or implantable hearing devices in children with congenital aural atresia: 25 years of our experience. International journal of pediatric otorhinolaryngology. 2015;79(7):975–9. 10.1016/j.ijporl.2015.03.031 25930173

[48] Hobson J.C., Roper A.J., Andrew R., Rothera M.P., Hill P., Green K.M. Complications of bone-anchored hearing aid implantation. The Journal of laryngology and otology. 2010;124(2):132–6. 10.1017/S0022215109991708 19968889

[49] Nelissen R.C., Mylanus E.A., Kunst H.P., Pennings R.J., Snik A.F., Hol M.K. A new bone-anchored hearing implant: short-term retrospective data on implant survival and subjective benefit. European archives of oto-rhino-laryngology: official journal of the European Federation of Oto-Rhino-Laryngological Societies (EUFOS): affiliated with the German Society for Oto-Rhino-Laryngology—Head and Neck Surgery. 2013;270(12):3019–25. 10.1007/s00405-013-2346-y 23358583

[50] Dun C.A., Faber H.T., de Wolf M.J., Mylanus E.A., Cremers C.W., Hol M.K. Assessment of more than 1,000 implanted percutaneous bone conduction devices: skin reactions and implant survival. Otology & neurotology: official publication of the American Otological Society, American Neurotology Society and European Academy of Otology and Neurotology. 2012;33(2):192–8. 10.1097/MAO.0b013e318241c0bf 22246385

[51] Chen S.Y., Mancuso D., Lalwani A.K. Skin Necrosis After Implantation With the BAHA Attract: A Case Report and Review of the Literature. Otology & neurotology: official publication of the American Otological Society, American Neurotology Society and European Academy of Otology and Neurotology. 2017;38(3):364–7. 10.1097/MAO.0000000000001327 28072655

[52] Baker S., Centric A., Chennupati S.K. Innovation in abutment-free bone-anchored hearing devices in children: Updated results and experience. International journal of pediatric otorhinolaryngology. 2015;79(10):1667–72. 10.1016/j.ijporl.2015.07.021 26279245

[53] Briggs R., Van Hasselt A., Luntz M., Goycoolea M., Wigren S., Weber P., et al Clinical performance of a new magnetic bone conduction hearing implant system: results from a prospective, multicenter, clinical investigation. Otology & neurotology: official publication of the American Otological Society, American Neurotology Society and European Academy of Otology and Neurotology. 2015;36(5):834–41. 10.1097/MAO.0000000000000712 25634465

[54] Carr S.D., Moraleda J., Procter V., Wright K., Ray J. Initial UK Experience With a Novel Magnetic Transcutaneous Bone Conduction Device. Otology & neurotology: official publication of the American Otological Society, American Neurotology Society and European Academy of Otology and Neurotology. 2015;36(8):1399–402. 10.1097/MAO.0000000000000830 26196208

[55] Clamp P.J., Briggs R.J. The Cochlear Baha 4 Attract System—design concepts, surgical technique and early clinical results. Expert review of medical devices. 2015;12(3):223–30. 10.1586/17434440.2015.990375 25496651

[56] Iseri M., Orhan K.S., Kara A., Durgut M., Ozturk M., Topdag M., et al A new transcutaneous bone anchored hearing device—the Baha(R) Attract System: the first experience in Turkey. Kulak burun bogaz ihtisas dergisi: KBB = Journal of ear, nose, and throat. 2014;24(2):59–64. 10.5606/kbbihtisas.2014.45143 24835899

[57] Iseri M., Orhan K.S., Tuncer U., Kara A., Durgut M., Guldiken Y., et al Transcutaneous Bone-anchored Hearing Aids Versus Percutaneous Ones: Multicenter Comparative Clinical Study. Otology & neurotology: official publication of the American Otological Society, American Neurotology Society and European Academy of Otology and Neurotology. 2015;36(5):849–53. 10.1097/MAO.0000000000000733 25730451

[58] Powell H.R., Rolfe A.M., Birman C.S. A Comparative Study of Audiologic Outcomes for Two Transcutaneous Bone-Anchored Hearing Devices. Otology & neurotology: official publication of the American Otological Society, American Neurotology Society and European Academy of Otology and Neurotology. 2015;36(9):1525–31. 10.1097/MAO.0000000000000842 26375976

[59] Centric A., Chennupati S.K. Abutment-free bone-anchored hearing devices in children: initial results and experience. International journal of pediatric otorhinolaryngology. 2014;78(5):875–8. 10.1016/j.ijporl.2014.02.004 24612554

[60] Denoyelle F., Coudert C., Thierry B., Parodi M., Mazzaschi O., Vicaut E., et al Hearing rehabilitation with the closed skin bone-anchored implant Sophono Alpha1: results of a prospective study in 15 children with ear atresia. International journal of pediatric otorhinolaryngology. 2015;79(3):382–7. 10.1016/j.ijporl.2014.12.032 25617189

[61] Denoyelle F., Leboulanger N., Coudert C., Mazzaschi O., Loundon N., Vicaut E., et al New closed skin bone-anchored implant: preliminary results in 6 children with ear atresia. Otology & neurotology: official publication of the American Otological Society, American Neurotology Society and European Academy of Otology and Neurotology. 2013;34(2):275–81. 10.1097/mao.0b013e31827d07f3 23444473

[62] Briggs R., et al, Clinical performance of a new magnetic bone conduction hearing implant system: results from a prospective, multicenter, clinical investigation. Otol Neurotol, 2015 36(5): p. 834–41. 10.1097/MAO.0000000000000712 25634465

[63] Carr S.D., et al, Initial UK Experience With a Novel Magnetic Transcutaneous Bone Conduction Device. Otol Neurotol, 2015 36(8): p. 1399–402. 10.1097/MAO.0000000000000830 26196208

[64] Hougaard D.D., et al, A multicenter study on objective and subjective benefits with a transcutaneous bone-anchored hearing aid device: first Nordic results. Eur Arch Otorhinolaryngol, 2017 274(8): p. 3011–3019. 10.1007/s00405-017-4614-8 28534117

[65] Iseri M., et al, Transcutaneous Bone-anchored Hearing Aids Versus Percutaneous Ones: Multicenter Comparative Clinical Study. Otol Neurotol, 2015 36(5): p. 849–53. 10.1097/MAO.0000000000000733 25730451

[66] Dimitriadis PA., Carrick S., Jaydip R., Intermediate outcomes of a transcutaneous bone conduction hearing device in a paediatric population. International Journal of Pediatric Otorhinolaryngology, 2017;94: 59–63. 10.1016/j.ijporl.2017.01.018 28167013

