# Supplementary material for: Active transcutaneous bone conduction hearing implants: Systematic review and meta-analysis
Source: PLoS One. 2019 Sep 16;14(9):e0221484. doi: 10.1371/journal.pone.0221484 (PMC6746395; doi:10.1371/journal.pone.0221484)
Supplement: S1 Table — (DOCX) [file pone.0221484.s001.docx]

S1 Table Demographic information on the study population. CHL conductive hearing loss, MHL mixed hearing loss, SSD single sided deafness, atBCI active transcutaneous bone conduction device, N/A information not available, ± standard deviation, M male, F female, COM chronic otitis media.

| Study | Location | Study design | Topic | Aetiology of HL | n total |
| --- | --- | --- | --- | --- | --- |
| Barbara et al. 2013 | Italy | prospective case series | atBCI in MHL | MHL, Cholesteatoma, COM | 4 |
| Sprinzl et al. 2013 | Germany, Austria | prospective case series | Short term efficacy and safety | cholesteatom, otosclerosis, COM, atresia auris, glomus tumor, chronic mastoiditis | 12 |
| Tsang 2013 | China | case report | first atBCI implantation in Asia | N/A | 1 |
| Ihler et al. 2014 | Germany | Retrosp. chart analysis, case series | audiological and safety outcomes with the atBCI | 2 CHL and 4 MHL, radical cavitiy, atresia, microtia | 6 |
| Lassaletta et al. 2014 | Spain | case report | audiological and safety outcomes with the atBCI | COM | 1 |
| Manrique et al. 2014 | Spain | prospective case series | audiological and safety outcomes with the atBCI under local- or general anesthesia | 4 M/CHL, COM , 1 SSD due to acoustic neuroma, | 5 |
| Matsumoto et al. 2014 | Japan | case series | pre-op planning | N/A | 3 |
| Mertens et al. 2014 | Nether-lands | case series | experimental method to measure Maximum Output and Dynamic | cholesteatoma and bilateral otosclerosis | 3 |
| Plontke et al. 2014 | Germany | prospective case series | Benefit of preoperative 3D planning tool | 4 chronic ear disease, 1 SSD, 1 malformation | 6 |
| Rahne et al. 2014 | Germany | Retrospective chart analysis, case series | functional outcome after atBCI implantation in adults and children | malformation, cholesteatom,  COM | 11 |
| Riss et al. 2014 | Austria |  |  | atresia of EAC | 23 |
| Schnabl et al. 2014 | Austria | case report | intra-op measurements with the atBCI | N/A | 2 |

| **S1 Table Demographic information on the study population.**  **(continued)** | | | | | |
| --- | --- | --- | --- | --- | --- |
| Study | Location | Study design | Topic | Aetiology of HL | n total |
| Wimmer et al. 2014 | Switzer-land | prospective case series | atBCI planning based on topographic bone thickness map | microtia/atresia, labyrinthitis, parotid tumor, mumps, COM, sudden hearing loss | 7 |
| Bianchin et al. 2015 | Italy | case series | Safety, Efficacy and QoL in 3 atBCI users | atresia auris, chronic otitis media with cholesteatoma and otosclerosis | 4 |
| Hassepass et al. 2015 | Germany |  |  | EAC atresia, Cholesteatom | 3 |
| Jovankovicova et al. 2015 | Slovak Republic | retrospective chart analysis | Congenital aural atresia and implantable hearing devices | aural atresia | 94 |
| Kim et al. 2015 | Korea | case report | atBCI treatment of bilateral cong. aural atresia | oval window atresia | 1 |
| Laske et al. 2015 | Switzerland | prospective case series | audiological and subjective outcomes of atBCI in SSD | lybyrinthitis (2), temp. bone fractures, viral infection (1), Menieres disease (1) | 9 |
| Rainsbury et al. 2015 | Canada | retrospective case series | pre-op headband assessment with post-op device performance | COM, ossicular fixation | 6 |
| Baum-gartner et al. 2016 | Austria | prospective case series | Safety and efficacy of the atBCI | atresia, COM, microtia, anotia, stenosis | 12 |
| Eberhard et al. 2016 | Denmark | prospective case series | Objective and subjective outcomes after atBCI implantation | COM, Otosclerosis, Cholesteatom, Mastoiditis, Vestibular Schwannoma | 12 |
| Gerdes et al. 2016 | Germany | prospective case series | Efficacy after atBCI implantation | N/A | 10 |
| Ihler et al. 2016 | Germany | prospective crossover study | atBCI versus Baha headband | cholesteatoma, CSOM, ME malformation | 8 |
| Lassaletta et al. 2016 | Spain, Argentina | prospective cohort study | postoperative pain | COM, dysgenesis, petrous bone fracture, otosclerosis | 27 |
| Law et al. 2016 | China | Case series | pre-operative planning of atBCI | COM, Otosclerosis, Atresia, Cholesteatoma, Sudden Idiopathic SNHL | 13 |

**S1 Table Demographic information on the study population.** **(continued)**

| Study | | Location | Study design | Topic | Aetiology of HL | n total |
| --- | --- | --- | --- | --- | --- | --- |
| Zernotti et al. 2016 | | Argentina | prospective comparative case series | Comparison of surgial and audiological outcomes of two BC devices | CAA, COM | 14 |
| Fan et al.  2017 | | China | prospective single-subject repeated measures | atBCI and auricle reconstruction | bilateral microtia-atresia, Jahrsdoerfer grading 5-7 | 12 |
| Monini et al. 2017 | Italy | | satisfaction survey | subjective satisfaction |  | 67 (4 with atBCI ) |
| Salcher et al. 2017 | Germany | | retrospective study, | atBCI in SSD | unknown, accident, skull fracture, Ménière's disease | 10 |
| Schmerber et al. 2017 | France, Belgium | | multicentric prospective study | atBCI safety and performance 1 yrs data | COM, atresia, cholesteat., oto-sclerosis, vestib-ular schwan-noma, trauma | 25 |
| Vyskocil et al. 2017 (n=35) | Austria | | retrospective case series | dura and sinus compression | Atresia, radical cavity cholesteat. tympanoscl. meningitis, oto-scl.,cong. SSD | 35-3 excl. |
| Vyskocil et al. 2017 (n=5) | Austria | | prospective single-subject repeated measures | localization with atBCI in unilateral CHL | microtia, atresia | 5 |
| Weiss et al. 2017 | Germany | | retrospective study | speech understanding and localization with the atBCI | radical cavity, tympanoplasty, otosclerosis, cholesteatoma, recurrent EO | 18 |
| Zhao et al. 2017 | China | | retrospective case series | atBCI in bilateral malformation | bilateral congenital malformation of outer and ME | 11 |
| Der et al.  2018 | Chile | | retrospective case series | middle fossa placement in children | bilateral microtia and congenital aural atresia | 24 |
| Kulasegarah et al. 2018 | New Zealand | | retrospective chart review | atBCI versus Ponto P on HB | atresia/ microtia | 10 |
| Ngui et al. 2018 | Malaysia | | prospective case series | children with cong. aural atresia | bi- unilateral canal atresia, | 6 |
| Zanetti et al. 2018 | Italy | | case report | atBCI in microtia with stapes ankylosis | microtia and stapes ankylosis | 2 |

| S1 Table Demographic information on the study population. CHL conductive hearing loss, MHL mixed hearing loss, SSD single sided deafness, atBCI active transcutaneous bone conduction device, N/A information not available, ± standard deviation, M male, F female, COM chronic otitis media. | | | | | | | |
| --- | --- | --- | --- | --- | --- | --- | --- |
| **Study** | **n total** | **CHL** | **MHL** | **SSD** | **Children <18 years** | **Age** | **Bilateral implants** |
|  |  |  |  |  |  | **(mean ± SD)** |  |
| Barbara et al. 2013 | 4 | 0 | 4 | 0 | 0 | 56 years (range 45 - 63) | 0 |
| Sprinzl et al. 2013 | 12 | 7 | 5 |  | 0 | 44 years (range 19-69 yrs) | 0 |
| Tsang 2013 | 1 | 1 | 0 | 0 | 0 | 57 years | 0 |
| Ihler et al. 2014 | 6 | 2 | 4 | 0 | 0 | 50.9±6.5 years (range 40-57.5) | 0 |
| Lassaletta et al. 2014 | 1 | 1 | 0 | 0 | 0 | 62 years | 0 |
| Manrique et al. 2014 | 5 | 2 | 2 | 1 | 0 | 56.2±8.1 years (range 43 - 64) | 0 |
| Matsumoto et al. 2014 | 3 | N/A | N/A | N/A | N/A | N/A | N/A |
| Mertens et al. 2014 | 3 | 3 | 0 | 0 | 0 | 50.3±12.7 years (range 43 - 65) | 0 |
| Plontke et al. 2014 | 6 | 3 | 1 | 2 | 1 | 47.7±22.2 years (range 10 - 76) | 0 |
| Rahne et al. 2014 | 11 | 4 | 6 | 1 | 3 | 37.7 years (range 5-76) | 0 |
| Riss et al. 2014 | 23 | 12 | N/A | N/A | 6 | 41 years (range 6-80) | N/A |
| Schnabl et al. 2014 | 2 | 2 | 0 | 0 | 0 |  | 0 |
| Wimmer et al. 2014 | 7 | 2 | 1 | 4 | 0 | 39.9±21.8 years (range 18 - 72) | 0 |
| Bianchin et al. 2015 | 4 | 1 | 1 | 1 | 0 | 31 | 0 |
| Hassepass et al. 2015 | 3 | 1 | 2 | 0 | 2 | 10.1 / 16.8 years | 0 |
| Jovankovicova et al. 2015 | 94 | 94 | 0 | 0 | 94 | N/A | 5 |
| Kim et al. 2015 | 1 | 1 | 0 | 0 | 1 | 8 | 0 |
| Laske et al. 2015 | 9 | 0 | 0 | 9 | 0 | 52±15 years | 0 |
|  |  |  |  |  |  |  |  |
| **S1 Table Demographic information on the study population. (continued)** | | | | | | | |
| **Study** | **n total** | **CHL** | **MHL** | **SSD** | **Children <18 years** | **Age** | **Bilateral implants** |
|  |  |  |  |  |  | **(mean ± SD)** |  |
| Pai et al. 2015 | 1 | N/A | N/A | N/A | N/A | 52 yrs | N/A |
| Rainsbury et al. 2015 | 6 | 6 | 0 | 0 | N/A | 37 ± 11.9 years | 1 |
| Baumgartner et al. 2016 | 12 | 12 | 0 | 0 | 12 | 5-17 years | 0 |
| Eberhard et al. 2016 | 12 | 5 | 3 | 4 | 0 | 45.1 years (range 20 - 69) | 0 |
| Gerdes et al. 2016 | 10 | N/A | N/A | N/A | N/A | BB: 51.1±17.7 years (range 26 - 75) | N/A |
|  |  |  |  |  |  | BAHA: 51.0±8.3-year (range, 41 - 64) |  |
| Ihler et al. 2016 | 8 | 6 | 2 | 0 | 0 | 38.8 ± 13.3 years | 0 |
| Lassaletta et al. 2016 | 27 | 24 (incl. MHL) | N/A | 3 | N/A | 44.4± 18.6 years | N/A |
| Law et al. 2016 | 13 | 12 | 0 | 1 | 0 | 41.8 years (range 18 - 59) |  |
| Zernotti et al. 2016 | 14 | N/A | N/A | N/A | N/A | 25.4 years (CI95 12-3-38.3) | N/A |
| Fan et al. 2017 | 12 | 12 | 0 | 0 | 12 | 11 ± 5 years (range 6-18 ) | 0 |
| Monini et al. 2017 | 67 (4 with Bonebridge) | 4 (incl. MHL) | N/A | 0 | 0 | 67.4 years (range 62.8-77.5) | N/A |
| Salcher et al. 2017 | 10 | 0 | 0 | 10 | 0 | 45 years (range 21-70) | N/A |
| Schmerber et al. 2017 | 25 | 7 | 6 | 12 | 0 | 44.1 ± 12.5 years (range 18 - 65) | 0 |
|  |  |  |  |  |  |  |  |
| **S1 Table Demographic information on the study population. (continued)** | | | | | | | |
| **Study** | **n total** | **CHL** | **MHL** | **SSD** | **Children <18 years** | **Age**  **(mean ± SD)** | **Bilateral implants** |
| Vyskocil et al. 2017 (n=35) | 35/38 analyzed (3 excluded) | 18 | 15 | 5 | 8 | 37 ± 21 years | 0 |
| Vyskocil et al. 2017 (n=5) | 5 | 5 | 0 | 0 | 2 | 35years (range 14 to 50) | 0 |
| Weiss et al. 2017 | 18 | 3 | 15 | 0 | 0 | 49 years (range 25-74) | 0 |
| Zhao et al. 2017 | 11 | 11 | 0 | 0 | N/A | 16.9 years (range 8-26) | 0 |
| Der et al. 2018 | 24 | 24 | 0 | 0 | 24 | 12 years (range 6-16) | 0 |
| Kulasegarah et al. 2018 | 10 | 10 | 0 | 0 | 10 | 9 years (range 5 to 15) | 3 |
| Ngui et al. 2018 | 6 | 6 | 0 | 0 | 6 | 15.8±2.6 years (range 15 - 18) | 0 |
| Zanetti et al. 2018 | 2 | 2 | 0 | 0 | 0 | 29/35 years | 0 |

| **S1 Table Surgical information on the study population.** (atBCI active transcutaneous bone conduction device, N/A information not available, ± standard deviation, M male, F female) | | | | | | |
| --- | --- | --- | --- | --- | --- | --- |
| **Study** | **Sex (M/F)** | **Follow-up** | **Lifts used** | **Sinus compression** | **Dura compression** | **Position of Bonebridge** |
| Barbara et al. 2013 | 3/1 | N/A | N/A | N/A | N/A | transmastoid, presigmoid and retrosigmoid approach (2/2) |
| Sprinzl et al. 2013 | 9/3 | 3 mo |  |  |  |  |
| Tsang 2013 | 0/1 | 1 mo |  |  |  |  |
| Ihler et al. 2014 | 1/5 | 3 months | N/A | N/A | N/A | N/A |
| Lassaletta et al. 2014 | h | 6 monts | N/A | N/A | N/A | retrosigmoidal |
| Manrique et al. 2014 | N/A | N/A | N/A | N/A | N/A | N/A |
| Matsumoto et al. 2014 | N/A | N/A | N/A | N/A | N/A | N/A |
| Mertens et al. 2014 | 1/2 | N/A | N/A | N/A | N/A | N/A |
| Plontke et al. 2014 | 3/3 | N/A | N/A | N/A | N/A | N/A |
| Rahne et al. 2014 | N/A | 5-24 months | N/A | N/A | N/A | 1 retrodimoidally, 10 sinus-duraangle, |
| Riss et al. 2014 | N/A | N/A |  |  |  |  |
| Schnabl et al. 2014 | N/A | first day after surgery | N/A | N/A | N/A | N/A |
| Wimmer et al. 2014 | 3/4 | 11 weeks | N/A | 5 | 1 | N/A |
| Bianchin et al. 2015 | 0/1 | 7.25 months (range 3–14) |  |  |  |  |
| Hassepass et al. 2015 | N/A | 6 mo |  |  |  |  |
| Jovankovicova et al. 2015 | 55/39 | N/A |  |  |  |  |
| Kim et al. 2015 | 0/1 | 3 mo |  |  |  |  |
| Laske et al. 2015 | 5/4 | 16 months (range, 11-22) | N/A | N/A | 1 | mastoid |
| Pai et al. 2015 | N/A | N/A | N/A | N/A | N/A | N/A |
| Rainsbury et al. 2015 | 2/4 | 4-6 weeks | N/A | N/A | N/A | N/A |
| Baumgartner et al. 2016 | 8/4 | 1 and 3 months | N/A | N/A | N/A | N/A |
|  |  |  |  |  |  |  |
|  |  |  |  |  |  |  |
| **S1 Table Surgical information on the study population.** | | | | | | |
| **Study** | **Sex (M/F)** | **Follow-up** | **Lifts used** | **Sinus compression** | **Dura compression** | **Position of Bonebridge** |
| Eberhard et al. 2016 |  | 7 months (range, 5 - 9) | N/A | 2 | 3 | 7 mastoid (sinodural angle), 5 retrosigmoid |
| Gerdes et al. 2016 |  | 8 weeks | N/A | N/A | N/A | N/A |
| Ihler et al. 2016 | N/A | 3 months | N/A | N/A | N/A | mastoid/ retrosigmoid |
| Lassaletta et al. 2016 | 9/18 | 15.2 (SD 10.0) months | Yes, 8/27 (30 %): 2-mm lifts in 6 cases, 3 mm in one case, one patient had 3 and 4 mm lifts on either side | 9/27 | 17/27 | 8/27 mastoid, 19/27 retrosigmoid |
| Law et al. 2016 | 6/7) | N/A | yes (9 /13)(4 TM and 5 RS)(2, 3, 4, 6mm) | 7 | 3 | 8 transmastoid and 5 retrosigmoid |
| Zernotti et al. 2016 | 7/7 | 18.7 mo (12-32 mo) |  |  |  |  |
| Fan et al. 2017 | 9/3 | 6 months | yes, min. one case not further specified | N/A | N/A | N/A |
| Monini et al. 2017 | 3/1 | min 1 year | N/A | N/A | N/A | N/A |
| Salcher et al. 2017 | 6/4 | 3 months | No | Yes | Yes (mean 2.1 mm, 0.7-2.8 mm, n= 7) | 10/10 retrosigmoid |
| Schmerber et al. 2017 | 8/17 | 12 months | No | No | 1/25 (no complication) | 21/25 sinodural, 3/25 retrosigmoid, 1/25 temporal line |
| Vyskocil et al. 2017 (n=35) | N/A | 29 (SD 13) months | Yes, 2 patients with exposed dura (2 and 3 mm) | 11/38 | 13/38 | 25/38 sinodural, 7/38 retrosigmoid, 4/38 middle fossa, 2/38 mix |
| Vyskocil et al. 2017 (n=5) | 3/2 | at least 14 weeks after first fitting | N/A | N/A | N/A | N/A |
| Weiss et al. 2017 | 6/12 | 12 months | N/A | N/A | N/A | N/A |
| Zhao et al. 2017 | 6/5 | 6 months | Yes (based on figure) | N/A | N/A | N/A |
| Der et al. 2018 | 11/13 | 17 months (2-36 months) | No | N/A | Yes? (not clearly described) | 24/24 middle-fossa |
| Kulasegarah et al. 2018 | 7/3 | N/A | in one bilateral patient 2 and 3 mm lifts | 5/13 | 3/13 | 13/13 mastoid |
| Ngui et al. 2018 | 4/2 | 6 months | N/A | N/A | N/A | 5 sinodural angle, 1retrosigmoid |
| Zanetti et al. 2018 | 1/1 | 36 months | N/A | No | No | mastoid |
