# Supplementary material for: Active transcutaneous bone conduction hearing implants: Systematic review and meta-analysis
Source: PLoS One. 2019 Sep 16;14(9):e0221484. doi: 10.1371/journal.pone.0221484 (PMC6746395; doi:10.1371/journal.pone.0221484)
Supplement: S2 Table — (DOCX) [file pone.0221484.s002.docx]

| Study | functional gain ( FG) [dB] | Speech understanding in quiet | Speech understanding in noise | Sound Localization |
| --- | --- | --- | --- | --- |
| Barbara et al. 2013 | 36.5±19.2 ( 14 - 53) | WRS at 50% speech reception thresholds (SRTs)  unaided: 77.5±17.1 (55 - 95)  aided: 41.3±14.4 (25 - 60)  **SRT improvement:** 36.3±17.5 (15 - 55) | N/A | N/A |
| Sprinzl 2013 | **PTA4 25 dB** from 55 to 30 dB HL # | Freiburger at 65 dB SPL improved from 14.2 % (± 18.1) to 92.9 % (± 6.9) at 3 mo OLSA SRT50% improved from 61.9 dB (± 8.6) to 36.6 dB (±8.8) at 3 mo | N/A | N/A |
| Tsang 2013 | PTA4 SF improved from  33 dB to 56 dB (data from Figure) = 23 dB | SRT improved by 32 dB in quiet in Cantonese Hearing in Noise Test. | In noise SNR improved from 8/4/8.5 dB to -0.1/-0.2/0.2 dB in S0N0/S0Npe/S0Nbe | N/A |
| Ihler et al. 2014 | 34.5±6.9 (0.5 -4.0 kHz) | Freiburger monosyllables at 65 dB SPL:  Unaided discrimination 16.7%±17.8%  aided discrimination 80.0 ±13.8%  **improvement 63.3%** | **Freiburger monosyllables at 65 dB SPL** (n=6)**:** unaided discrimination 8.3%±13.3%; aided discrimination 45.8 ± 14.0%; **improvement 37.5%** | N/A |
| Lassaletta et al. 2014 | 42 dB (68 to 26 dB HL) | SDS at 65 dB: + 85 % (from 0 to 85 %) | N/A | N/A |
| Manrique et al. 2014 | 35.6±12.1  (SSD: 17.5) | Disyllabic word discrimination  unaided 66.2±11.4%,  aided 86.2±7.5; **Improv.: 20.0±8.1 *(p=0.016)*** | N/A | N/A |
| Matsumoto et al. 2014 | N/A | N/A | N/A | N/A |
| Mertens et al. 2014 | N/A | N/A | N/A | N/A |
| Plontke et al. 2014 | N/A | WRS @65dB  unaided: 12.5±19.4 %  aided: 91.7±5.2% | **SRT HINT (S0N0)**  unaided: -2.7±3.1, aided: -3.4±2.4  **SRT HINT (S90N90)**  unaided: -1.5±2.6, aided: -6.4±4.4  **improvement -7.2±3.2** | Angle detection error (degrees)  unaided: 60.3±36.4  aided: 36.0±34.5 |
| Rahne 2015 | Effective gain (unaided AC-aided SF): PTA4 33.4 dB (± 5.7 dB) | WRS @65 dB unaided 10±19 % at 65 dB SPL (Freiburger)  aided: 87.5 ± 8.9 %  **Improvement : 77.5 %** | **SRT in noise S0N0** unaided -2.3 dB SNR (± 2.6 dB) aided: -3.3 dB SNR (± 1.8 dB) **SRT in noise in S90N-90** unaided: 1.3 dB SNR (± 2.1 dB) aided: 6.1 dB SNR (± 3.1 dB) | Angle detection error (degrees)  unaided: 37±38  aided: 27±20 |

S2 Table. Audiological outcomes with the atBCI (AC air conduction, BC bone conduction, N/A not available/not reported, SPL speech presentation level, ± standard deviation, SNR signal to noise ratio, PTA4 (pure tone average over freq. 0.5, 1, 2, 4kHz), WRS word recognition score, BCHA bone conduction hearing aid, SRT speech reception threshold, SDS speech discrimination score, # data extracted from figure)

| **S2 Table Audiological outcomes with the atBCI (continued)** | | | | |
| --- | --- | --- | --- | --- |
| Study | functional gain ( FG) [dB] | Speech understanding in quiet | Speech understanding in noise | Sound Localization |
| Riss 2014 | 28.8 dB (± 16.1 dB) all CHL: 32.5dB HL (± 14.3)  MHL: 24.7dB HL (± 18.4) SSD: 27.2dB HL (± 13.4) | WRS (Freiburger) at 65 dB SPL: from 4.6 % (± 7.4) to 53.7 % (± 23.0) at 80 dB SPL: from 33.0 % (± 30.0) to 77.5 % (± 19.0) | N/A | N/A |
| Schnabl et al. 2014 | PTA4: Pat 1: 40 and Pat 1: 55 dB | N/A | N/A | N/A |
| Wimmer et al. 2014 | PTA 4: 29.7 dB | N/A | N/A | N/A |
| Bianchin 2015 | 33.1±7.6 effective gain 42.3dB | SRT 50 % improved from 63±5.8 to 33±2.9 dB disyllabic word test @65 dB improved from 3.3% to 100 % | N/A | N/A |
| Hassepass et al. 2015 | CHL: 35 dB HL  M/CHL: 33 dB HL | **Freiburger WRS@65 dB:**  M/CHL unaided: 0 % CHL unaided: 0%  M/CHL aided: 47.5 % CHL aided: 25%  **SRT 50 %:**  M/CHL unaided: 53.5 CHL unaided: 70  M/CHL aided: 29 CHL aided: 33  **Improvement: M/CHL: 24.5 CHL: 37** | N/A | N/A |
| Jovankovi-cova 2015 | PTA4: Surgery: 12 dB (from 66 to 54 dB HL); Hearing implant: 37 dB (from 65 to 29 dB HL) | N/A | N/A | N/A |
| Kim 2015 | 24 dB | **SRT improv. 48 dB** (from 66 to 18 dB SPL) SDS from 92 % at MCL 90 dB to 100 % at MCL 50 dB | N/A | N/A |
| Laske et al. 2015 | N/A | N/A | **OLSA (S0N0)**  unaided: -2.82, aided: -2.14  **OLSA (SCLN0)**  unaided: -7.27, aided: -6.52  **OLSA S_device_ N0**  unaided: -1.73, aided: -3.38 | N/A |
| Pai et al. 2015 | N/A | N/A | N/A | N/A |
| Rainsbury 2015 | PTA (0.5, 1, 2, 3 kHz) 42.5 dB | SRT unaided 70 dB (67.5-70 dB) | N/A | N/A |
|  |  | SRT aided 25 dB (20-27.5 dB) |  |  |

| **S2 Table Audiological outcomes with the atBCI**  **(continued)** | | | | |
| --- | --- | --- | --- | --- |
| Study | functional gain ( FG) [dB] | Speech understanding in quiet | Speech understanding in noise | Sound Localization |
| Baum-gartner 2016 | 46.9 dB | Goettinger Kindersprachtest/Freiburger **Monosyllables Test/OLSA WRS in quiet** pre-OP: 14.5 % (± 21.6) 1 month post-OP: 67.2 % (± 17.9) 3 months post-OP: 82.1 % (± 12.1)  **OLSA and OLKISA: SRT50% in quiet:** pre-OP: 72.7 dB SPL (± 5.9) 1 month post-OP: 52.5 dB SPL (± 8.2) 3 month post-OP: 45.2.5 dB SPL (± 6.9) | N/A | N/A |
| Eberhard et al. 2016 | Overall mean: 23.4±14.9  M/CHL mean: 25.0±18.2  SSD mean: 20.3±4.7 | SDS monosyllable word list (Dantale)@50, 65, 80 dB SPL: | SRT50%Danish Hagerman sentences (Dantale II)@70dB  unaided: 75.6%±10.6  aided: 70.4%±7.4  **improvement:**  SSD unaided: 67.3%±2.7  SSD aided: 67.3%±3.2 | N/A |
|  |  | unaided: 28.2%±34.8, 50.6%±44, 78.0%±25.5 |  |  |
|  |  | aided: 68.4%±29.6, 84.1%±24.9, 90.1%±11.2 |  |  |
|  |  | **improvement: 40.2%±32.6, 33.6%± 41.0, 12.1%±21.0** |  |  |
|  |  | SSD unaided: 54.7%± 21.7, no outcomes reported for 65 dB and 80 dB SPL |  |  |
|  |  | SSD aided: 70.7%±26.1 no outcomes reported for 65 dB and 80 dB SPL; |  |  |
|  |  | **SSD improvement: 16.0%±4.4** |  |  |
| Gerdes et al. 2016 | atBCI : 27.± 9.7 dB  BAHA: 26.3±11.2 dB | WRS at 65 dB SPL unaided mean atBCI : 11 % and BAHA: 9,5%  atBCI aided: 89% (, 65 -100%)  BAHA aided: 86,5% (, 60 - 100%)  **Improvement atBCI : 78%; BAHA: 77%** | SNR S0N0: unaided mean atBCI : 3.5 BAHA: 5.1 dB SNR  atBCI : -2.8 dB (, -5.9 to 1:7)  BAHA: -2.5 dB (, -4.5 to -0.2)  **Improvment in noise: atBCI : 6.3 dB SNR BAHA: 7.7dB SNR** | N/A |
| Ihler et al. 2016 | atBCI: 28.9 (± 4.2) dB  Baha Headband: 15.8 (± 2.7) dB | N/A | OLSA in 65 dB SPL noise:  unaided: 4.6 (± 4.2) dB SNR ( -2.0 to 11.2)  Baha headband: -3.3 (± 7.2) dB SNR) ( -18.5 to 4.8)  atBCI : -1.2 (± 4.0) dB SNR ( -8.4 to 4.2) | N/A |
| Lassaletta et al. 2016 | 500-4000 Hz:  C/MHL: 26.6 dB  SSD 38.8 (± 4.4) dB | disyllabic SDS in quiet at 65 dB SPL (C/MHL):  unaided 56.7 (± 38.6) %  atBCI 97.5 (± 6.1) % | SSD subjects: SRT in noise (disyllabic words)  noise at 55 dB SPL: from unaided 35 to 25 dB HL aided  noise at 65 dB SPL: from 40 dB unaided to 30 dB with atBCI | N/A |

| **S2 Table Audiological outcomes with the atBCI**  **(continued)** | | | | |
| --- | --- | --- | --- | --- |
| Study | functional gain ( FG) [dB] | Speech understanding in quiet | Speech understanding in noise | Sound Localization |
| Law et al. 2016 | TM gorup: 33.0±12.0 | N/A | N/A | N/A |
|  | RS group: 29.0±16.0 |  |  |  |
| Zernotti 2016 | Functional gain: atBCI 40 (± 13) dB Sophono 34 (± 16) dB | N/A | N/A | N/A |
| Fan et al. 2017 | FG (PTA4)  atBCI: 34 dB (from 55 dB HL to 21 dB HL), Softband: 24 dB (from 55 to 31 dB HL) | Disyllabic test at 65 dB SPL:  atBCI 47.5 % improvement (from 46 ±11 % to 94 ± 2 %)  Softband: 34 % (from 46 to 80 %) | N/A | N/A |
| Monini et al. 2017 | N/A | N/A | N/A | N/A |
| Salcher et al. 2017 | PTA4 in sound field improved from 64 to 28 dB HL with contralateral plugged and muffled (p <0.001) | WRS (Freiburger at 65 dB SPL) improved from 0 to 80 %  OLSA in quiet (speech implanted side): SRT from 34.52 dB SPL to 32.26 dB SPL (p = 0.003) | OLSA in 65 dB SPL noise: | N/A |
|  |  |  | speech front, noise contralateral: from -1.77 to -3.08 dB SNR (p = 0.005) |  |
|  |  |  | speech implanted side, noise contralateral: from 1.86 to -0.64 dB SNR (p = 0.003) |  |
| Schmerber et al. 2017 | C/MHL functional gain 26.1 (±13.7) dB HL  C/MHL aided SF PTA4 25.1 (± 11.3) dB HL | WRS at 65 dB SPL (Fournier word list):  C/MHL unaided 74 (± 35) %  C/MHL atBCI: 95 % (± 15) (p = 0.034) | WRS at 65 dB SPL (Fournier word list) in 55 dB SPL noise:  C/MHL unaided 62 % (± 40 %)  C/MHL atBCI: 89 % (± 25 %) (p = 0.022)  **SSD:**Speech SSD side, noise NH side: unaided 51.30±3.90 dB SPL#, aided 48.99± 4.47 dB SPL#;  S0 Nssd: unaided 48.16±4.81 dB SPL#, aided 48.34± 4.95 dB SPL | N/A |
| Vyskocil et al. 2017 (n=5) | Average functional gain 25.7 dB (± 2.6 dB) | Median WRS (Freiburger at 65 dB SPL) i  unaided median: 10 % (10-20 %)  aided median: 80 % (55-80 %)  **Median improvement 60 % points (55-70 %).** | OLSA in noise (no information on noise level)  S0N0 unaided 11.5 (10.2-15.3) dB SNR to aided 2.9 (-0.9 to 4) dB SNR  **median improvement: 11.1 dB (7.5 - 12.4) dB**  S90N-90 from unaided 7.4 (5.1 - 13.0) dB SNR to -1.6 (-3.7 to 1.6) dB SNR  **median improvement: 9-0 (8.8 - 11.4) dB** | Median RMS error angles:  Unaided 18.8 to 88.0  Aided 14.2 to 32.6  Change 3.6 to 61.5 |

| **S2 Table Audiological outcomes with the atBCI**  **(continued)** | | | | | |
| --- | --- | --- | --- | --- | --- |
| Study | functional gain ( FG) [dB] | Speech understanding in quiet | Speech understanding in noise | Sound Localization |  |
| Vyskocil et al. 2017 (n=35) | 26.9 (± 15.6) dB | Median Freiburger monosyllables at 65 dB SPL:  unaided median: 5 % (0-20 %)  aided median: 65 % (60-83.75 %)  **median** i**mprovement** 60 % (41.25 - 67.75%) | N/A | N/A |  |
| Weiss et al. 2017 | (PTA4 AC vs PTA4 SF aided): 29.3 (± 20.7) dB | Freiburger at 65 dB SPL (numbers) from 54.4 % (± 39.7 %) to 100 % (± 0 %); with the atBCI  Freiburger at 65 dB SPL (monosyl. words) from 44.7 % ( 0-90%) to 70.6 % ( 45 to 85 %) with HA and to 91.3 % ( 55-100 %) with atBCI .  **Average improv.** compared to best aided 20.7 % (p = 0.028) | OLSA in 65 dB SPL speech and adaptive noise, N = 11:  SRT improved from -3.8 dB SNR ( -5.67 to 5.8 dB SNR) to -5.2 dB SNR ( -6.3 to -0.6 dB SNR) (p<0.005) | score 11.4° ( 3.3-30.8°)  aided 13.7° (1.8-43.8°)  no significant difference |  |
| Zhao et al. 2017 *(data from abstract – ms in chinese)* | <12 yrs: from 63/60 dB Hl to 36/40 dB HL  >12 yrs: from 62 (± 6.5) dB HL to 34.2 (± 5.3) dB HL | **MLNT at 70 dB SPL:**  Monosyl.: easy 10/30 % to 20/50 %; difficult 10/10 % to 20/20 %  Disyl.: easy 70/80 % to 90/100%; difficult 75/75 to 80-90%  **MSTM at 65 dB SPL**  Monosyl. 10 (± 7) % to 57 (± 5) %; Disyl. 36 (± 6) % to 65 (± 8) %; MSTM at 80 dB SPL; monosyllables 28 (± 9) % to 70 (± 10) %; Disyllables 40 (± 11) 5 to 90 (± 10) % | N/A | N/A |  |
| Der et al. 2018 | with atBCI at activation 31.0 dB HL (95%CI 28.2-33.8)  with atBCI after 1 mo 25.2 dB HL (95%CI 23.5-26.9)  1 mo **41.3 dB** HL (95%CI 37.7-44.9, p < 0.001) | speech recognition (speech at 65 dB SPL, monosyllables, disyllables or spondees and known terms in Spanish)  before surgery 29.4 % (95%CI 25.2-34.6)  with BC hearing aid 78.9 % (95%CI 73.5-84.4)  with atBCI: at 1 month 96.4 % (95%CI 92.7-100.2) | N/A | N/A |  |
| Kulasegarah et al. 2018 | Aided PTA4:  atBCI: 25.5 (± 3.0) dB HL  BCHA 26.5 (± 3.2) dB HL | CVC words in quiet (N= 9):  unaided 78.3 dB HL / 86.8 %  atBCI: 38.5 dB HL / 83.2 %  BCHA: 40 dB HL / 79.9 % | QuickSIN and BKB-SIN (four talker noise at 65-70 dB HL)  unaided 3.7 to 10.5; atBCI 0.2 to 1.2 dB; BCHA 0.8 to 6.5 dB SNR | N/A |  |
| Ngui et al. 2018 | 46.3±12.6 ( 31 - 61 dB) | N/A | N/A | N/A |  |
| Zanetti et al. 2018 | Pat.1: PTA4 40.5 dB  Pat. 2: PTA4 38.0 dB | 100 % WRS in sound field in quiet:  Pat.1: at 85 dB HL; postop at 25 dB HL  Pat.2: at 65 dB HL; postop at 20 dB HL | **WRS in noise:**  **Pat.1:** unaided max. WRS 55 % to 88 % at 40 dB HL with atBCI ; **Pat.2:** unaided max. WRS 60 % to 85 % at 50 dB HL with atBCI (after 3 yrs) | N/A |  |
