# Supplementary material for: Active transcutaneous bone conduction hearing implants: Systematic review and meta-analysis
Source: PLoS One. 2019 Sep 16;14(9):e0221484. doi: 10.1371/journal.pone.0221484 (PMC6746395; doi:10.1371/journal.pone.0221484)
Supplement: S3 Table — (DOCX) [file pone.0221484.s003.docx]

**S3 Table. Subjective outcomes with the active transcutaneous bone conduction implant (atBCI)** (# data extracted from figure)

| **Study** | **n subjects** | **Subjective outcomes** |
| --- | --- | --- |
| Barbara et al. 2013 | 4 | No subjective outcomes reported |
| Sprinzl et al. 2013 | 12 | HDSS: 79% (, 49 - 99) |
| Tsang 2013 | 1 | No subjective outcomes reported |
| Ihler et al. 2014 | 6 | GBI: total score 32.4±13.5, general 45.8±14.9, social 8.3±13.9 , 2.8±12.5 |
| Lassaletta et al. 2014 | 1 | No subjective outcomes reported |
| Manrique et al. 2014 | 5 | No subjective outcomes reported |
| Matsumoto et al. 2014 | 3 | No subjective outcomes reported |
| Mertens et al. 2014 | 3 | No subjective outcomes reported |
| Plontke et al. 2014 | 6 | No subjective outcomes reported |
| Rahne et al. 2014 | 11 | No subjective outcomes reported |
| Riss et al. 2014 | 23 | No subjective outcomes reported |
| Schnabl et al. 2014 | 2 | No subjective outcomes reported |
| Wimmer et al. 2014 | 7 | No subjective outcomes reported |
| Bianchin et al. 2015 | 4 | GHSI unaided: |
|  |  | Total:18.05±27.07, general: 33.33±42.18, social: 47.83±14.41, physical: 64.58±14.23, |
|  |  | GBI aided: |
|  |  | Total: 40.97±5.26, general: 58.33±6.80, social: 20.83±24.99, physical: 20.83±24.99, |
| Hassepass et al. 2015 | 3 | No subjective outcomes reported |
| Jovankovicova et al. 2015 | 94 | No subjective outcomes reported |
| Kim et al. 2015 | 1 | No subjective outcomes reported |
| Laske et al. 2015 | 6 (out of 9) | atBCI SS:patients had good benefit from the device with variations among subjects of 4.5 points. One subject reported no benefit (Overall mean score extracted from graph: 2.2)  SSQ-B: Part1 score 1.5, Part2 score 0.6, Part3 score 1.0 |
| Pai et al. 2015 | 1 | No subjective outcomes reported |
| **S3 Table. Subjective outcomes with the active transcutaneous bone conduction implant (atBCI) (continued)** | | |
| **Study** | **n**  **subjects** | **Subjective outcomes** |
| Rainsbury et al. 2015 | 6 | No subjective outcomes reported |
| Baumgartner et al. 2016 | 12 | No subjective outcomes reported |
| Eberhard et al. 2016 | 12 | IOI-HA all: 3.7±1.0 |
|  |  | IOI-HA SSD: 2.9±0.7 |
|  |  | SSQ all: overall score 5.1±1.6, hearing score 4.9±2.2, sound localisation score 3.4±2.4, quality of hearing 6.5±1.4 |
|  |  | SSQ SSD: overall score 4.5±1.5 |
| Gerdes et al. 2016 | 10 | APHAB:  EC: unaided: atBCI : 44±29%; BAHA: 74±20%, aided: atBCI : 10±8%; BA: 16±10% (mean values from text)  EC: unaided: atBCI : 50; BAHA: 81; aided: atBCI : 8; BAHA: 13 (MEDIAN values from graph)  BN: unaided: atBCI : 80; BAHA: 79; aided: atBCI : 19; BAHA: 31 (MEDIAN values from graph)  RV: unaided: atBCI : 75; BAHA: 82 aided: atBCI : 17; BAHA: 29 (MEDIAN values from graph)  AV: unaided: atBCI : 8; BAHA: 8; aided: atBCI : 15; BAHA: 46 (MEDIAN values from graph) |
| Ihler et al. 2016 | 8 | GBI: atBCI total score 38.5 (± 16.5); Baha Headband total score -6.9 (± 25.9), 5/8 negative total score  APHAB: atBCI global scale 15.2 (± 14.9), Baha Headband global scale 28.2 (± 27.8). Benefit of atBCI over headband was 28.3 (± 28.9) for EC, 22.7 (± 25.4) for RV, 27.3 (± 24.7) for BN and 6.5 (± 18.3) for AV. Overall benefit was 12.9 (± 24.9) |
| Lassaletta et al. 2016 | 27 | Headache Impact Test (HIT-6): mean preoperative pain severity score of 42.6 and mean postoperative score of 41.8  Brief Pain Inventory (BPI): mean score changed from 0.6 to 0.9 postoperatively, inference score changed from 0.1 to 0.3 |
| Law et al. 2016 | 13 | No subjective outcomes reported |
| Schmerber et al. 2016 | 25 | APHAB: a tendency to perform better with the atBCI compared to the unaided condition was observed for the subscales Ease of Communication, Background Noise and Reverberation. Aversiveness of Sound was greater with the atBCI , but not significant.  GBI: C/MHL: total score 30#, general score 40 (± 27.1), social score 10 (± 27.3), physical score 8 (± 25.2)  GBI: S±: total score 15#, general score 20 (± 25.6), social score 2 (± 9.5), physical score 0 (± 7.9)  IOI-HA: C/MHL: 12/12 wear device all day, 9/12 high benefit, 11/12 less activity limitation, 9/12 very high level of satisfaction, 10/12 no restricted in participation, 10/12 impact on social relationship, 10/12 improvement in quality of life  IOI-HA: S±: 6/9 use device all day, 5/9 high benefit, 6/9 less activity limitation, 6/9 very high level of satisfaction, 6/9 not restricted in participation 5/9 improvement in quality of life |
| Zernotti et al. 2016 | 14 | No subjective outcomes reported |

| **S3 Table. Subjective outcomes with the active transcutaneous bone conduction implant (atBCI) (continued)** | | | |
| --- | --- | --- | --- |
| **Study** | | **n subjects** | **Subjective outcomes** |
| Fan et al. 2017 | 12 | | APHAB#: ease of communication from 75 to 10 % (unaided to atBCI aided), background noise from 50 to 30 %, reverberation from 50 % to 20 %, aversiveness of sound from -10 to-55 %  GCBI: final score with atBCI was 39.6 ± 3.6  IOI-HA: mean score from 4 to 5 on all items |
| Monini et al. 2017 | 4 | | Only data for atBCI (n = 4) are extracted:  Mean total GBI score: 35.21 (social health 40, general health 30, physical health 22)  Visual analogue scale: quality of sound increased from 5.5 to 8.1, quality of life VAS >6 in n = 4  APHAB: mean total percentage score from 58% with conventional hearing aid to 23 % with atBCI (all domains significantly different with p> 0.05, except background noise domain) |
| Salcher et al. 2017 | 10 | | APHAB#: Ease of Communication changed from 28 (± 27 ) to 11 (± 11) (p = 0.039); Background Noise from unaided 67 (± 25) to 50 (± 27) (p = 0.195), Reverberation from unaided 46 (± 15) to 27 (± 15) (p = 0.008), Aversiveness from unaided 35 (± 32) to 36 (± 33) (p = 0.688)  atBCI SS: average benefit 2.8 compared to unaided (p < 0.001); speech in quiet (p = 0.008), distant speaker (p = 0.004), reverberant room (p=0.008), overall (p = 0.002) |
| Vyskocil et al. 2017 | 5 | | No subjective outcomes reported |
| Vyskocil et al. 2017 | 35 | | No subjective outcomes reported |
| Weiss et al. 2017 | 17 | | Modified atBCI SS (scale -5 to +5): average score 2.7, no negative average score; worst rating for hearing with the atBCI in noise (score 1.7) and background sound and in live rooms (score 1.4); overall satisfaction very high 3.6 |
| Zhao et al. 2017 | 11 | | No subjective outcomes reported |
| Der et al. 2018 | 24 | | No subjective outcomes reported |
| Kulasegarah et al. 2018 | 10 | | PEACH questionnaire (n=7): 85.7 scored within "typical performance" for overall, quiet and noise |
| Ngui et al. 2018 | 6 | | HDSS: 95.5±2.7 (, 91 - 98) |
| Zanetti et al. 2018 | 2 | | APHAB (Pat1/Pat2) : ease of communication improved from 16.3/25 to 8.3/8.3, background noise from 33.3/25.0 to 12.0/12.0, aversiveness of sound from 18.5/30.0 to 1.0/11.0, reverberation from 31.3/31.0 to 30.8/30.2 |
|  |  |  | SSQ (Pat1/Pat2): speech subdomain changed from 1.4/2.0 to 7.6/7.3, spatial from 0.5/2.4 to 3.2/6.5, qualities from 2.6/1.8 to 6.8/7.5 |
