# Supplementary material for: Active transcutaneous bone conduction hearing implants: Systematic review and meta-analysis
Source: PLoS One. 2019 Sep 16;14(9):e0221484. doi: 10.1371/journal.pone.0221484 (PMC6746395; doi:10.1371/journal.pone.0221484)
Supplement: S4 Table — (DOCX) [file pone.0221484.s004.docx]

**S4 Table. Safety outcomes** (F/U follow up, # number of)

| **Study and population properties** | | | | | | | | | | |  |
| --- | --- | --- | --- | --- | --- | --- | --- | --- | --- | --- | --- |
| Study | mean follow-up (months) | SD follow-up | # patients | # implanted ears | # ears excluded from analysis | # ears lost -to-F/U | Person-years | % of ears with complications | Incidence rate | **# of ears final** |  |
| Lassaletta et al. 2014 | 6 | ns | 1 | 1 | 0 | 0 | 0,5 | 0,00 | 0,00 | 1 |  |
| Bianchin et al. 2015 | 7,25 | 4,64 | 4 | 4 | 0 | 0 | 2,4 | 0,00 | 0,00 | 4 |  |
| Lassaletta et al. 2016 | 15,2 | 10 | 27 | 27 | 0 | 0 | 34,2 | 0,00 | 0,00 | 27 |  |
| Zanetti et al. 2017 | 36 | ns | 2 | 2 | 0 | 0 | 6,0 | 0,00 | 0,00 | 2 |  |
| Weiss et al. 2017 | 12 | ns | 18 | 18 | 0 | 0 | 18,0 | 0,00 | 0,00 | 18 |  |
| Schmerber et al. 2016 | 12 | ns | 28 | 28 | 3 | 2 | 23,0 | 0,04 | 0,04 | 23 |  |
| Eberhard et al. 2016 | 7,01 | 1,11 | 12 | 12 | 0 | 0 | 7,0 | 0,08 | 0,14 | 12 |  |
| Der et al. 2018 | 17 | ns | 24 | 24 | 0 | 0 | 34,0 | 0,21 | 0,15 | 24 |  |
| Laskeet al. 2015 | 16 | ns | 9 | 9 | 0 | 0 | 12,0 | 0,22 | 0,17 | 9 |  |
| Ihler et al. 2014 | 8,5 | 2,2 | 6 | 6 | 0 | 0 | 4,3 | 0,17 | 0,24 | 6 |  |
| Ngui et al. 2018 | 6 | ns | 6 | 6 | 0 | 0 | 3,0 | 0,17 | 0,33 | 6 |  |
| Hassepass et al. 2015 | 6 | ns | 3 | 3 | 0 | 0 | 1,5 | 0,33 | 0,67 | 3 |  |
| Sprinzl et al. 2013 | 3 | ns | 12 | 12 | 0 | 0 | 3,0 | 0,33 | 1,33 | 12 |  |
| Barbara et al. 2013 | ns | ns | 4 | 4 | 0 | 0 |  | 0,00 |  | 4 |  |
| Tsang et al. 2013 | ns | ns | 1 | 1 | 0 | 0 |  | 1,00 |  | 1 |  |
| Manrique et al. 2014 | ns | ns | 5 | 5 | 0 | 0 |  | 0,00 |  | 5 |  |
| Rahne et al. 2014 | ns | 5 to 24 | 11 | 11 | 0 | 0 |  | 0,09 |  | 11 |  |
| Riss et al. 2014 | ns | ns | 24 | 24 | 0 | 1 |  | 0,09 |  | 23 |  |
| Wimmer et al. 2014 | ns | ns | 7 | 7 | 0 | 0 |  | 0,00 |  | 7 |  |
| Kim et al. 2015 | ns | ns | 1 | 1 | 0 | 0 |  | 0,00 |  | 1 |  |
| Law et al. 2015 | ns | ns | 13 | 13 | 0 | 0 |  | 0,00 |  | 13 |  |
| Matsumoto et al. 2015 | ns | ns | 3 | 3 | 0 | 0 |  | 0,00 |  | 3 |  |
| Baumgartner et al. 2016 | ns | ns | 12 | 12 | 0 | ns |  | 0,25 |  | 12 |  |
| Pai et al. 2016 | ns | ns | 1 | 1 | 0 | 0 |  | 0,00 |  | 1 |  |
| Zernotti et al. 2016 | ns | ns | 10 | 10 | 0 | 0 |  | 0,10 |  | 10 |  |
| Vyskocil et al. 2017 | ns | 2 to 50 | 38 | 38 | 2 | 1 |  | 0,03 |  | 35 |  |
| Kulasegarah et al. 2018 | ns | ns | 10 | 13 | 0 | 0 |  | 0,15 |  | 13 |  |
| % of all patients experienced complication(-s): | | | **9,4** | **F/U dependent** incidence rate overall: | | | **0,107** |  | | |  |
| % of all patients experienced only **minor** complications: | | | **7,7** | **F/U dependent** incidence rate **minor:** | | | **0,101** | -> **1 out of 10** subjects experiences **minor AEs** (per year F/U) | | |  |
| % of all patients experienced **major** complications: | | | **1,7** |  |  |  |  |  |  |  |  |
| % of all patients experienced **no complications:** | | | **88,5** | **F/U dependent** incidence rate **major:** | | | **0,007** | -> **7 out of 1000** subjects experience **major AEs** (per year F/U) | | |  |
| % of all complications were solved: | | | **100,0** |  |  |  |  |  |  |  |  |
