# Supplementary material for: Active transcutaneous bone conduction hearing implants: Systematic review and meta-analysis
Source: PLoS One. 2019 Sep 16;14(9):e0221484. doi: 10.1371/journal.pone.0221484 (PMC6746395; doi:10.1371/journal.pone.0221484)
Supplement: S5 Table — (DOCX) [file pone.0221484.s005.docx]

**S5 Table. Safety outcome categories** (AE adverse event, RS revision surgery; # number of)

| **Study** | **ears with AE** | | | | | | **ears with RS or expl.** | | **major** complications (leading to RS or expl.) | | | | **minor** complications | | | | | | | | | | | | | | | | **Non-user** |
| --- | --- | --- | --- | --- | --- | --- | --- | --- | --- | --- | --- | --- | --- | --- | --- | --- | --- | --- | --- | --- | --- | --- | --- | --- | --- | --- | --- | --- | --- |
|  | **# of ears final** | # earswith complication | # ears with ONLY minor complication | # ears with major complication | # ears with no complication | # ears with solved complication | # of revision surgeries | # of explantations | patients' request to remo-ve atBCI (Psychological, Radiotherapy, MRI etc…) | ischemia of the reconstructed earlobe | chronic fibrosing mastoiditis | out of indication ->non-user | Technical problem with FM | AP handling issues | herpangina infection | headaches | mild wound pain | recurrent cholesteotoma | tinnitus | hematoma ear | Infection | Skin edema or erythema | Skin/wound infection | prolonged wound healing | itching around the AP site | seroma at implant site | Skin inflammation secondary to magnet strength | dizziness or vertigo | no or limited benefit, |
| Lassaletta et al. 2014 | 1 | 0 | 0 | 0 | 1 | 0 |  |  |  |  |  |  |  |  |  |  |  |  |  |  |  |  |  |  |  |  |  |  |  |
| Bianchin et al. 2015 | 4 | 0 | 0 | 0 | 4 | 0 |  |  |  |  |  |  |  |  |  |  |  |  |  |  |  |  |  |  |  |  |  |  |  |
| Lassaletta et al. 2016 | 27 | 0 | 0 | 0 | 27 | 0 |  |  |  |  |  |  |  |  |  |  |  |  |  |  |  |  |  |  |  |  |  |  |  |
| Zanetti et al. 2017 | 2 | 0 | 0 | 0 | 2 | 0 |  |  |  |  |  |  |  |  |  |  |  |  |  |  |  |  |  |  |  |  |  |  |  |
| Weiss et al. 2017 | 18 | 0 | 0 | 0 | 18 | 0 |  |  |  |  |  |  |  |  |  |  |  |  |  |  |  |  |  |  |  |  |  |  |  |
| Schmerber et al. 2016 | 23 | 1 | 0 | 1 | 22 | 1 |  | 1 | 1 |  |  |  |  |  |  |  |  |  |  |  |  |  |  |  |  |  |  |  |  |
| Eberhard et al. 2016 | 12 | 1 | 1 | 0 | 11 | 1 |  |  |  |  |  |  |  |  |  |  |  |  |  |  | 1 |  |  |  |  |  |  |  |  |
| Der et al. 2018 | 24 | 5 | 5 | 0 | 19 | 5 |  |  |  |  |  |  |  |  |  |  |  |  |  | 1 |  | 4 |  |  |  |  |  |  |  |
| Laske et al. 2015 | 9 | 2 | 2 | 0 | 7 | 2 |  |  |  |  |  |  |  |  |  |  |  |  |  |  | 1 |  |  | 1 |  |  |  |  |  |
| Ihler et al. 2014 | 6 | 1 | 1 | 0 | 5 | 1 |  |  |  |  |  |  |  |  |  |  |  |  |  |  |  |  |  |  |  |  |  |  |  |
| Ngui et al. 2018 | 6 | 1 | 1 | 0 | 5 | 1 |  |  |  |  |  |  |  |  |  |  |  |  |  |  |  |  | 1 |  |  |  |  |  |  |
| Hassepass et al. 2015 | 3 | 1 | 1 | 0 | 2 | 1 |  |  |  |  |  |  |  |  |  |  |  |  |  | 1 |  |  |  |  |  |  |  |  |  |
| Sprinzl et al. 2013 | 12 | 4 | 4 | 0 | 8 | 4 |  |  |  |  |  |  |  |  |  | 1 |  |  | 1 |  | 1 |  |  |  |  | 1 |  |  |  |
| Barbara et al. 2013 | 4 | 0 | 0 | 0 | 4 | 0 |  |  |  |  |  |  |  |  |  |  |  |  |  |  |  |  |  |  |  |  |  |  |  |
| Tsang et al. 2013 | 1 | 1 | 1 | 0 | 0 | 1 |  |  |  |  |  |  |  |  |  |  | 1 |  |  |  |  |  |  |  |  |  |  | 1 |  |
| Manrique et al. 2014 | 5 | 0 | 0 | 0 | 5 | 0 |  |  |  |  |  |  |  |  |  |  |  |  |  |  |  |  |  |  |  |  |  |  |  |
| Rahne et al. 2014 | 11 | 1 | 0 | 1 | 10 | 1 | 1 |  |  |  | 1 |  |  |  |  |  |  |  |  |  |  |  |  |  |  |  |  |  |  |
| Riss et al. 2014 | 23 | 2 | 0 | 2 | 21 | 2 |  | 1 |  |  |  | 2 |  |  |  |  |  |  |  |  |  |  |  |  |  |  |  |  | 2 |
| Wimmer et al. 2014 | 7 | 0 | 0 | 0 | 7 | 0 |  |  |  |  |  |  |  |  |  |  |  |  |  |  |  |  |  |  |  |  |  |  |  |
| Kim et al. 2015 | 1 | 0 | 0 | 0 | 1 | 0 |  |  |  |  |  |  |  |  |  |  |  |  |  |  |  |  |  |  |  |  |  |  |  |
| Law et al. 2015 | 13 | 0 | 0 | 0 | 13 | 0 |  |  |  |  |  |  |  |  |  |  |  |  |  |  |  |  |  |  |  |  |  |  |  |
| Matsumoto et al. 2015 | 3 | 0 | 0 | 0 | 3 | 0 |  |  |  |  |  |  |  |  |  |  |  |  |  |  |  |  |  |  |  |  |  |  |  |
| Baumgartner et al. 2016 | 12 | 3 | 2 | 1 | 9 | 3 | 1 |  |  | 1 |  |  |  |  | 1 |  |  |  |  |  |  |  |  |  | 1 |  |  |  |  |
| Pai et al. 2016 | 1 | 0 | 0 | 0 | 1 | 0 |  |  |  |  |  |  |  |  |  |  |  |  |  |  |  |  |  |  |  |  |  |  |  |
| Zernotti et al. 2016 | 10 | 1 | 1 | 0 | 9 | 1 |  |  |  |  |  |  |  |  |  |  | 1 |  |  |  |  |  |  |  |  |  |  |  |  |
| Vyskocil et al. 2017 | 35 | 1 | 1 | 0 | 34 | 1 |  |  |  |  |  |  |  |  |  |  |  |  |  |  |  |  | 1 |  |  |  |  |  |  |
| Kulasegarah et al. 2018 | 13 | 2 | 2 | 0 | 11 | 2 |  |  |  |  |  |  | 1 | 8 |  |  |  |  |  |  |  |  | 1 |  |  |  | 1 |  |  |
